# Bacterial-Chromatin Structural Proteins Regulate the Bimodal Expression of the Locus of Enterocyte Effacement (LEE) Pathogenicity Island in Enteropathogenic *Escherichia coli*

**DOI:** 10.1128/mBio.00773-17

**Published:** 2017-08-08

**Authors:** Hervé Leh, Ahmad Khodr, Marie-Christine Bouger, Bianca Sclavi, Sylvie Rimsky, Stéphanie Bury-Moné

**Affiliations:** a LBPA, Université Paris Saclay, CNRS, ENS Paris Saclay, Cachan, France; b Center for Interdisciplinary Research in Biology (CIRB), Collège de France, CNRS, INSERM, PSL Research University, Paris, France; c Institute for Integrative Biology of the Cell (I2BC), CEA, CNRS, Université Paris Sud, Université Paris-Saclay, Gif-Sur-Yvette, France; National Cancer Institute

## Abstract

In enteropathogenic *Escherichia coli* (EPEC), the locus of enterocyte effacement (LEE) encodes a type 3 secretion system (T3SS) essential for pathogenesis. This pathogenicity island comprises five major operons (*LEE1* to *LEE5*), with the *LEE5* operon encoding T3SS effectors involved in the intimate adherence of bacteria to enterocytes. The first operon, *LEE1*, encodes Ler (LEE-encoded regulator), an H-NS (nucleoid structuring protein) paralog that alleviates the LEE H-NS silencing. We observed that the *LEE5* and *LEE1* promoters present a bimodal expression pattern, depending on environmental stimuli. One key regulator of bimodal *LEE1* and *LEE5* expression is *ler* expression, which fluctuates in response to different growth conditions. Under conditions *in vitro* considered to be equivalent to nonoptimal conditions for virulence, the opposing regulatory effects of H-NS and Ler can lead to the emergence of two bacterial subpopulations. H-NS and Ler share nucleation binding sites in the *LEE5* promoter region, but H-NS binding results in local DNA structural modifications distinct from those generated through Ler binding, at least *in vitro*. Thus, we show how two nucleoid-binding proteins can contribute to the epigenetic regulation of bacterial virulence and lead to opposing bacterial fates. This finding implicates for the first time bacterial-chromatin structural proteins in the bimodal regulation of gene expression.

## IMPORTANCE

Gene expression stochasticity is an emerging phenomenon in microbiology. In certain contexts, gene expression stochasticity can shape bacterial epigenetic regulation. In enteropathogenic *Escherichia coli* (EPEC), the interplay between H-NS (a nucleoid structuring protein) and Ler (an H-NS paralog) is required for bimodal *LEE5* and *LEE1* expression, leading to the emergence of two bacterial subpopulations (with low and high states of expression). The two proteins share mutual nucleation binding sites in the *LEE5* promoter region. *In vitro*, the binding of H-NS to the *LEE5* promoter results in local structural modifications of DNA distinct from those generated through Ler binding. Furthermore, *ler* expression is a key parameter modulating the variability of the proportions of bacterial subpopulations. Accordingly, modulating the production of Ler into a nonpathogenic *E. coli* strain reproduces the bimodal expression of *LEE5*. Finally, this study illustrates how two nucleoid-binding proteins can reshape the epigenetic regulation of bacterial virulence.

## INTRODUCTION

Bacterial population heterogeneity improves bacterial survival in different environments and gives rise to adaptation strategies within complex communities (1–5). Nongenetic phenotypic heterogeneity primarily results from cellular responses to random environmental signals, cell aging, and stochastic gene expression. Stochastic gene expression contributes to bacterial epigenetics (i.e., heritable phenotypic heterogeneity without genetic mutation) and collective behaviors, supporting the concept of bacterial multicellularity (5–7).

In pathogenic bacteria, stochastic gene expression can lead to distinct virulent states (8) or persistence (9, 10) or heterogeneity in host immune responses (11). Under virulence-inducing conditions, bimodal expression patterns have been reported for several pathogenicity factors. These factors include expression of type 1 pili by *Streptococcus pneumoniae* (12) and type III secretion system (T3SS) by the phytopathogenic bacterium *Dickeya dadantii* (13) or *Pseudomonas aeruginosa* (14).

During *Salmonella enterica* serotype Typhimurium infection, division of labor occurs (15), with only some cells producing the T3SS. However, the fraction of bacteria producing SPI-1 T3SS acquires a growth penalty, resulting in loss of fitness (8). Most SPI-1-expressing bacteria die inside host cells, generating inflammation (16). In turn, in the gut lumen, inflammation confers a selective advantage to the mainly non-SPI-1-expressing *Salmonella* over the microbiota and thereby promotes the stability of virulence in the evolutionary context (15, 17). Similarly, phenotypically T3SS-expressing- and non-T3SS-expressing bacteria coexist within the *P. aeruginosa* population in a murine model of acute pneumonia, suggesting that non-T3SS-expressing bacteria behave as cheaters, taking advantage of T3SS-expressing bacteria (14). Taken together, these studies highlight the importance of gene expression stochasticity to ensure the necessary phenotypes required for successful infection and survival.

In attaching/effacing (A/E) pathogens, such as enteropathogenic *Escherichia coli* (EPEC) and enterohemorrhagic *Escherichia coli* (EHEC), the expression of T3SS is central to pathogenesis and is associated with the locus of enterocyte effacement (LEE) pathogenicity island. LEE is a horizontally acquired AT-rich DNA locus and comprises 41 genes arranged in ﬁve polycistronic operons (designated *LEE1* to *LEE5*) (18–20). The expression of all LEE genes is silenced by H-NS, an abundant nucleoid-associated protein. H-NS is a xenogeneic silencer that acts as a repressor of gene expression in elements recently acquired horizontally (21, 22). Indeed, H-NS preferentially blocks transcription at these AT-rich acquired loci, facilitating foreign DNA incorporation into the chromosome. In addition to promoters of their own genes, AT-rich regions contain sequences that mimic polymerase-binding sites. Thus, transcription start sites have been mapped to unexpected locations in bacterial genomes, including the noncoding strand. H-NS also acts to silence these elements. Hence, a key function of H-NS is to ensure transcriptional specificity (23). H-NS organizes bacterial chromatin by binding to regions *in vivo* as long as 1,500 bp (24), forming nucleoprotein filaments organized in either stiffened or bridged DNA conformations depending on the presence of Mg^2+^ (25–29). H-NS-bound regions are associated with low or no transcriptional activity (22, 30–32). At promoters, silencing by H-NS is often alleviated by H-NS antagonists that interfere with the H-NS–DNA complex structure, with or without concomitant displacement of H-NS (33, 34). Among these antagonists, Ler, the first protein produced from *LEE* under the control of the products of the *perABC* operon, is an H-NS paralog. Ler relieves H-NS silencing specifically at *LEE* promoters and a few other targets (20, 35). Recently, a growth rate bimodality, mediated by a hysteretic memory switch, was reported for EPEC (36). This bimodality results in the coexistence of nonvirulent and hypervirulent subpopulations. The hypervirulent subpopulation continues to express virulence after several generations of growth under nonactivating conditions. The main regulators of this hysteretic switch are the products of the *perABC* operon. Ler itself is not involved (36). This heterogeneity has been proposed to reflect a bet-hedging strategy (36). In this case, a subset of the cell population presents a phenotype considered nonoptimal or nonadapted that may be advantageous if environmental conditions change (e.g., sudden stress, rapid return to a previous situation). For example, in *E. coli*, such strategy has been reported for SOS genes and colicin expression (37, 38).

The *LEE*5 promoter (*P*_*LEE5*_) controls the operon encoding the adhesin intimin (*eae*), its receptor (*tir*), and a chaperone (*cesT*). The intimin and Tir proteins are major virulence factors (39). The aim of the present study is to explore whether the opposing regulatory effects of Ler and H-NS on T3SS expression in EPEC at the individual cell level can be involved in a bimodal population pattern.

Here, we describe the bimodal expression pattern of *P*_*LEE5*_ under growth conditions generally considered mimicking conditions nonoptimal for virulence. This expression pattern is controlled by the interplay of H-NS and Ler. We show that H-NS and Ler, binding at the same nucleation DNA motif, induce different nucleoprotein structures in the isolated *P*_*LEE5*_. Finally, we observe that under different environmental conditions, the level of Ler expression is a key element controlling the bimodality of *LEE5* expression under different environmental conditions. Thus, the balance between H-NS silencing and Ler antisilencing activities generates nongenetic variability.

## RESULTS

### The expression from the *LEE5* promoter is bimodal in exponential phase.

Classically, infections of epithelial cells with EPEC are assayed in Dulbecco’s modified Eagle’s medium (DMEM). Indeed, the expression of EPEC virulence is generally considered to be active when grown in DMEM at 37°C. In such “activating” conditions, most virulence genes are expressed but not in Luria-Bertani liquid medium (LB) (see “Media” in Materials and Methods), “nonactivating” conditions (40–43).

In order to explore a potential population phenotypic heterogeneity, we assessed *P*_*LEE5*_ (i.e., normally expressing intimin and Tir) activity in EPEC in these activating and nonactivating conditions. We wished to explore the heterogeneity of *LEE5* expression at the individual cell level under these two conditions, since it might reflect either bet-hedging or division of labor strategies. In the case of bet-hedging, we could expect, for example, the presence of a subpopulation of* LEE5*-expressing bacteria in nonactivating conditions (LB). In contrast, a division of labor strategy could be indicated by bimodal expression of *LEE5* in activating condition (DMEM).

**FIG 1:**
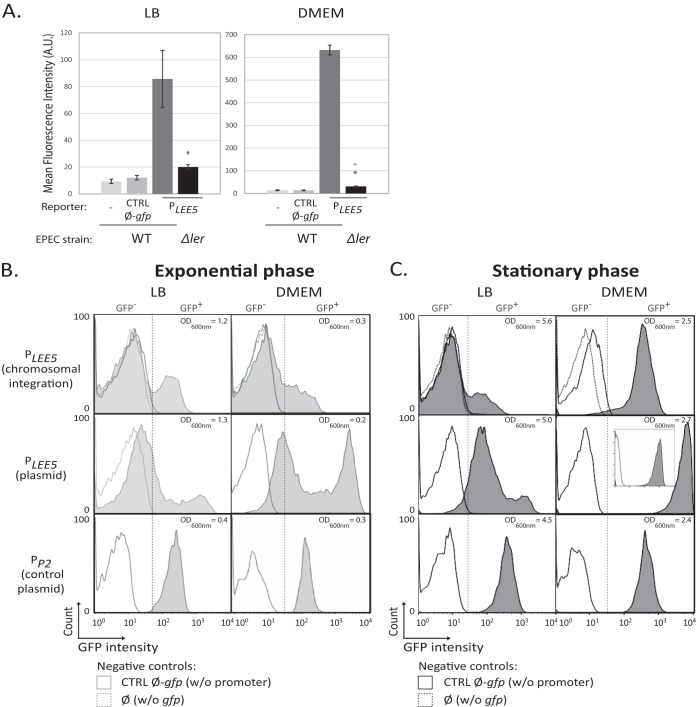
Bimodality of *LEE5* expression. (A) Analysis of *LEE5* promoter activity from a single chromosomal copy in WT EPEC at the population level. WT EPEC and Δ*ler* EPEC strains containing the *gfp* reporter gene under the control of the *LEE5* promoter (*P*_*LEE5*_) inserted at the *attB*_Phi80_ phage site were cultured with agitation at 37°C in LB and DMEM. In the stationary phase (24 h after 1:1,000 dilution of a culture grown overnight in LB), the mean fluorescence (in arbitrary units [A.U.]) of the entire bacterial population was determined. Values are means ± standard errors (error bars) from 5 and 3 independent experiments performed in LB and DMEM, respectively. Statistical differences between the WT and its isogenic mutant with the cassette and between the Δ*ler* mutant and the controls (CTRL Ø-*gfp*) are indicated with black and gray asterisks, respectively (based on a Student one-paired *t*-test; *, *P* < 0.05). (B and C) Analysis of *LEE5* and constitutive phage promoter activities at the individual cell level. WT EPEC strains containing the *gfp* reporter gene under the control of the *LEE5* promoter inserted at the *attB*_Phi80_ phage site [*P*_*LEE5*_ (chromosomal insertion)], the pKK-P_*LEE5*_-*gfp* plasmid [*P*_*LEE5*_ (plasmid)], or the pKK-Prom*P2*-*gfp* plasmid [*P*_*P2*_ (control plasmid)] were cultured under the same condition as in panel A. In exponential phase (3 h after 1:1,000 dilution of an LB-overnight culture [B]) or in the stationary phase (24 h after inoculation [C]), the mean fluorescence of individual bacteria was determined using flow cytometry analysis. In parallel, the optical density at 600 nm (OD_600nm_) was determined. The results from one representative experiment of three independent experiments are presented for each condition. The positions of the GFP-negative (GFP^−^) and GFP-positive (GFP^+^) subpopulations are separated by a dashed line. The basal bacterial fluorescence was measured using either the WT strain without (w/o) the reporter cassette (Ø) or WT strain containing the pKK-*gfp* promoter-less* gfp* plasmid (CTRL Ø-gfp).

To do so, we introduced a *gfp* reporter under the control of *P*_*LEE5*_ as a single copy on the EPEC chromosome at the *attB*_Phi80_ phage site and performed flow cytometry analysis (Fig. 1). Mean fluorescent measurement of the whole population (Fig. 1A) confirmed that the upregulation of *P*_*LEE5*_-*gfp* by Ler is eightfold higher in DMEM than in LB.

At the individual cell level in exponential phase, bimodal *P*_*LEE5*_ expression was observed in both LB and DMEM (Fig. 1B). Two subpopulations of bacteria were observed, bacteria expressing green fluorescent protein (GFP^+^) and bacteria expressing very low levels of GFP or not expressing GFP (GFP^−^). The peak corresponding to GFP^+^ bacteria is visible only as a shoulder, presumably because the low cell fluorescence is closed to the sensitivity threshold. To amplify the signal and to confirm the presence of a bimodal phenotype in the cell population, we used a low-copy-number plasmid reporter (≈10 copies per cell) carrying the *P_LEE5_-gfp* cassette (44, 45). This allowed us to clearly observe two subpopulations of cells expressing GFP either at a low level (“low state,” with a distribution that differs slightly from the negative control without promoter) or expressing GFP at a high level (“high state”). The latter subpopulation displays a mean fluorescence intensity, as anticipated from the gene dosage effect, increased by 1 log unit compared to the subpopulation of GFP^+^ bacteria containing one chromosomal insertion (Fig. 1B). Using this reporter system thus yields a better discrimination of the different populations and confirms the bimodal population pattern in exponential phase in both LB and DMEM (Fig. 1B).

For a control, a wild-type (WT) EPEC strain expressing *gfp* from the constitutive T5 phage P2 promoter was analyzed. The GFP expression pattern was unimodal throughout the bacterial population in all growth conditions (Fig. 1B; see also Fig. S1 in the supplemental material).

10.1128/mBio.00773-17.1 FIG S1  Phage promoter activity in WT, Ler, or H-NS-deficient EPEC strains. (Top) Medium effect on a T5 phage promoter P2 activity in WT, Ler, or H-NS-deficient EPEC strains. (Bottom) Doubling time and ratio of maximal density reached in stationary phase after growth of the EPEC strains in Glc-CAA-M9 medium. The WT EPEC, Δ*ler*, and Δ*hns* single and double mutant strains containing the pKK-*P_P2_-gfp* plasmid and the WT strain containing the pKK-*gfp* plasmid (CTRL Ø-*gfp*) were grown under agitation at 37°C in LB and DMEM. In the stationary phase (24 h), GFP expression was determined by flow cytometry analysis. The results of the single-cell analysis are the results of one representative experiment (of three independent experiments). The pre-early promoter of phage T5 (*P*_*P2*_), a constitutively expressed promoter, was used as a reference. The top two graphs indicate that its activity does not vary significantly either in WT or Δ*ler* and Δ*hns* single mutant strains. The mean fluorescence of the double mutant strain is higher than that of the single mutant strains. (Bottom) The Δ*ler* Δ*hns* double mutant strain presents a significant lower generation time. The biomass yield reached in stationary phase by this Δ*ler* Δ*hns* mutant is twofold lower than the yields for the WT, Δ*ler*, and Δ*hns* single mutant strains. Thus, the growth penalty of the Δ*ler* Δ*hns* double mutant results in an apparent increase in GFP expression by reducing the dilution of fluorescence occurring during cell division (see Fig. S2) (S. Klumpp and T. Hwa, Curr Opin Biotechnol 28:96–102, 2014, https://doi.org/10.1016/j.copbio.2014.01.001). Then, the increase of GFP expression under the control of *LEE* promoters was not considered relevant in the double mutant strain. These results indicate that the GFP expression from a low-copy-number plasmid is unimodal regardless of the medium and Ler and H-NS expression. Download FIG S1, PDF file, 0.6 MB. Copyright © 2017 Leh et al. 2017 Leh et al. This content is distributed under the terms of the Creative Commons Attribution 4.0 International license.

### *LEE5* promoter expression progressively involves all the cells in activating conditions.

In stationary-phase cultures expressing *P*_*LEE5*_-*gfp*, two population patterns could be observed: a unimodal distribution, corresponding to the high state (growth in DMEM), and a bimodal distribution (growth in LB) (Fig. 1C). Under these conditions, the level of fluorescence in the cells results from GFP accumulation throughout the whole growth phase, since GFP is stable over the time of the experiment. We monitored the dilution of the GFP fluorescence to an undetectable level through cell division by shifting the culture to a nonpermissive temperature for LEE expression (Fig. S2) (20). We concluded that for the bimodal distribution in LB, the low-state subpopulation corresponds to bacteria that either never activated *P*_*LEE5*_ or activated it transiently during exponential phase. In the case of the unimodal population in DMEM, GFP accumulation thus indicates that all cells had expressed *LEE5* at a high level in the experiment. Therefore, in activating conditions (DMEM), the switch on of *P*_*LEE5*_ is progressively spreading to the whole population.

10.1128/mBio.00773-17.2 FIG S2  Kinetic of fluorescence decline by dilution during cell division. The WT EPEC strain containing the pKK-*P_LEE5_-gfp* plasmid was grown overnight in DMEM at 37°C, diluted at 1:1,000 in DMEM, and grown at 30°C. Bacterial growth and fluorescence at 30°C were monitored for 290 min by time-lapse microscopy using a Nikon Eclipse Ti microscope with a 100× immersion objective. Fluorescence intensity was quantified using ImageJ software. The trendline equation of fluorescence decline and the coefficient of determination (*R*^2^) are indicated. Bacteria were grown at 37°C in DMEM to allow maximal expression of GFP under the control of *P*_*LEE5*_ (optimal virulence expression condition). Then, the bacteria were diluted and shifted to 30°C. At 30°C, GFP production is blocked as a result of *P*_*LEE5*_ repression. As bacteria divide, the GFP fluorescence declines as a result of the intracellular GFP dilution. Download FIG S2, PDF file, 0.4 MB. Copyright © 2017 Leh et al. 2017 Leh et al. This content is distributed under the terms of the Creative Commons Attribution 4.0 International license.

To explore the hysteresis of the high state (i.e., its maintenance when the conditions that initially upregulate the promoter are not occurring anymore), we tested different culture inoculation conditions (Fig. S3). Notably, we observed that a unimodal cell population (inoculated from a culture in activating conditions, i.e., DMEM) reinoculated in fresh DMEM displayed a bimodal population pattern in exponential phase. This indicates that a resettable phenotypic switch controls the activation of *LEE5* expression and that this phenotypic bimodal expression of *LEE5* is not hysteretic.

10.1128/mBio.00773-17.3 FIG S3  Variation of *LEE5* activity pattern depending on the medium and growth phase of inoculating overnight culture. (A) Precultures of the WT EPEC strain containing pKK-*P*_*LEE5*_-*gfp* (*P*_*LEE5*_) or pKK-*gfp* (CTRL Ø-*gfp*) plasmid were cultured under agitation at 37°C in LB. Inoculations of two subcultures were done. The exponential-phase inoculum was done by diluting the preculture at 1:100 (black line). On the other hand, the stationary-phase inoculum was done by diluting the preculture grown overnight at 1:1000 (gray lines). Both cultures were grown for 3 h after dilution, and GFP expression was determined by flow cytometry analysis. The results shown are from one experiment representative of two independent experiments. (B) The experiments were done keeping the same culture conditions as in panel A except that both precultures and cultures were grown in DMEM. The results shown are from one experiment representative of two independent experiments. The results show that the bimodal pattern is independent of both the inoculum growth phase and the inoculum medium. Notably, bacteria grown overnight in DMEM and used to inoculate a fresh DMEM present a bimodal pattern of* LEE5* expression in exponential phase. This result indicates that *LEE5* expression activation is triggered by a resettable phenotypic switch. Download FIG S3, PDF file, 0.3 MB. Copyright © 2017 Leh et al. 2017 Leh et al. This content is distributed under the terms of the Creative Commons Attribution 4.0 International license.

Further analyses described below were all carried out in stationary phase, where the difference between LB (bimodal distribution) and DMEM (unimodal distribution) with respect to the pattern of *LEE5* expression is observable (Fig. 1C).

### The level of *LEE5* promoter expression varies with the composition of the growth medium.

**FIG 2:**
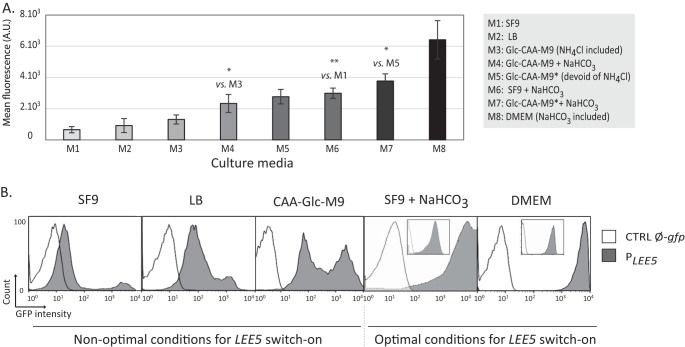
Impact of environmental conditions on *LEE5* expression using a plasmid reporter. (A) *LEE5* promoter activity in WT EPEC at the population level in different environmental conditions. The WT strain containing the pKK-*P*_*LEE5*_-*gfp* plasmid was grown at 37°C in eight different media (M1 to M8). *P*_*LEE5*_ activity was analyzed in stationary phase by cytometry analysis in synthetic media with sodium bicarbonate (DMEM) or without sodium bicarbonate (Glc-CAA-M9, Glc-CAA-M9*, and SF9). Standard Glc-CAA-M9 contains 18.4 mM NH_4_Cl. Glc-CAA-M9 devoid of NH_4_Cl is designated Glc-CAA-M9*. Where indicated, the media were supplemented with 45 mM NaHCO_3_. Values are means ± standard errors from 3, 4, 6, 5, 8, 3, 8, and 6 independent experiments for cells grown in the M1 to M8 media, respectively. Some significant differences by Student’s two-paired *t* test are indicated by asterisks as follows: *, *P* < 0.05; **, *P* < 0.01. (B) Flow cytometry analysis of *gfp* expression from the *LEE5* promoter at the individual cell level. The WT EPEC containing the pKK-*PLEE5*-*gfp* (*P*_*LEE5*_) or pKK-*gfp* (CTRL Ø-*gfp*) plasmid construct were grown with agitation at 37°C in CAA-Glc-M9, LB, SF9 (supplemented with 45 mM bicarbonate or not supplemented with bicarbonate) and DMEM. GFP expression was measured in stationary phase (24 h). For DMEM and SF9 supplemented with bicarbonate conditions, a second acquisition was performed with modified settings in order to assess the entire bacterial population (insets). The results depicted show the results of one representative experiment from at least three independent experiments.

To assess the impact of environmental conditions on the pattern of *LEE5* expression and to mimic gastrointestinal repression or induction signals (39), we monitored *LEE5* expression at stationary phase in various media. Notably, we tested the effect of ammonium chloride or sodium bicarbonate (the former acts as an inhibitor and the latter acts as an activator of *LEE5* expression). Figure 2 shows the flow cytometry analysis of the WT EPEC with a *P_LEE5_-gfp* reporter grown in eight different media.

The mean fluorescence of the whole cell population (Fig. 2A) displayed a continuum of values for *LEE5* expression levels according to the medium type. This apparent continuous variation reflects the average fluorescence of the entire population resulting from the distribution between the two subpopulations (Fig. 2B). Indeed, depending on the composition of the medium, we again observed two patterns of *LEE5* expression: a unimodal distribution corresponding to the high state (growth in DMEM or SF9 medium supplemented with sodium bicarbonate), and a bimodal distribution (growth in LB, SF9 medium, and CAA-Glc-M9 medium [M9 base with 0.5% Casamino Acids and 0.4% glucose] [see “Media” in Materials and Methods]) (Fig. 2B).

Altogether, our results indicate that describing media as activating and nonactivating does not adequately reflect the complexity of *LEE5* expression. Henceforth, we shall therefore use the term “nonoptimal conditions” for *P*_*LEE5*_-repressing conditions at the whole-population level (e.g., LB, SF9, and CAA-Glc-M9), conditions where *LEE5* expression is low in most bacteria and high only in a small fraction of them. We will use the term “optimal conditions” when *LEE5* expression was upregulated in all bacteria (e.g., DMEM, SF9 containing bicarbonate).

In conclusion, *LEE5* expression was activated in all bacteria grown under optimal conditions but only in a subpopulation under nonoptimal conditions. This finding shows that under conditions classically considered to be repressive for LEE expression (40–43), a small subpopulation is expressing *LEE5* at high levels, suggesting a potential bet-hedging strategy.

### The nucleoid-associated proteins Ler and H-NS are essential regulators of *LEE5* bimodality.

**FIG 3:**
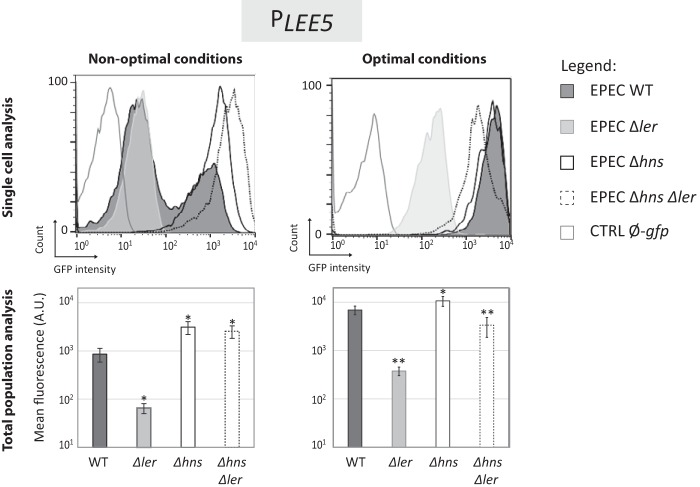
Bimodal expression from *LEE5* depends on H-NS and Ler. The WT EPEC, Δ*ler*, and Δ*hns* single and double mutant strains containing the pKK-*P*_*LEE5*_-*gfp* plasmid were grown at 37°C in LB (nonoptimal conditions) and DMEM (optimal conditions). The WT EPEC strain containing the pKK-*gfp* plasmid (CTRL Ø-*gfp*) was used as a negative control. (Top) In stationary phase, GFP expression was determined using flow cytometry analysis. The results of a representative experiment are shown. (Bottom) The corresponding mean fluorescence of the whole bacterial population (± standard error) measured in at least five independent experiments for each condition. Values that are significantly different from the WT value by Student’s two-paired *t* test are indicated by asterisks as follows: *, *P* < 0.05; **, *P* < 0.01.

To assess the roles of Ler and H-NS in the bimodal expression of *LEE5*, *gfp* expression under the control of *P*_*LEE5*_ was monitored in WT EPEC and Δ*ler*, Δ*hns* single or double mutant EPEC strains grown in nonoptimal (LB) and optimal (DMEM) media (Fig. 3). In the absence of Ler and the presence of H-NS, only one population of bacteria was observed, and the peak was in the low state, confirming that Ler is required in some way for *LEE5* activation in both LB and DMEM. Because Ler is required for virulence (46, 47), we suggest that the low state likely corresponds to nonvirulent bacteria. This hypothesis is in accordance with the identification of a hypervirulent bacterial subpopulation that expressed Ler and T3SS at high levels (36). These experiments suggested a direct link between the level of Ler expression and virulence in an EPEC subpopulation.

We observed that the mean fluorescence of the Δ*ler* bacterial population was 1 log unit higher in DMEM than in LB, indicating that the medium composition affects the basal activity of *P*_*LEE5*_, independent of Ler (Fig. 3). In the absence of H-NS, Ler was no longer required for *P*_*LEE5*_ activation, confirming that the main role of Ler on this promoter is to relieve H-NS silencing. In DMEM, deletion of* hns* has no apparent effect on *LEE5* expression, indicating that in this growth medium, there is no repression by H-NS (due to Ler antisilencing activity). Interestingly, in LB medium, genetic inactivation of H-NS led to an upregulated unimodal distribution of fluorescence in the bacterial population, with all cells being in the high state (Fig. 3). Taken together, these observations indicate that both the Ler and H-NS proteins are required for the bimodal expression of *LEE5*.

### Ler and H-NS bind common sites on the *LEE5* promoter but affect differently the local DNA structure *in vitro.*

**FIG 4:**
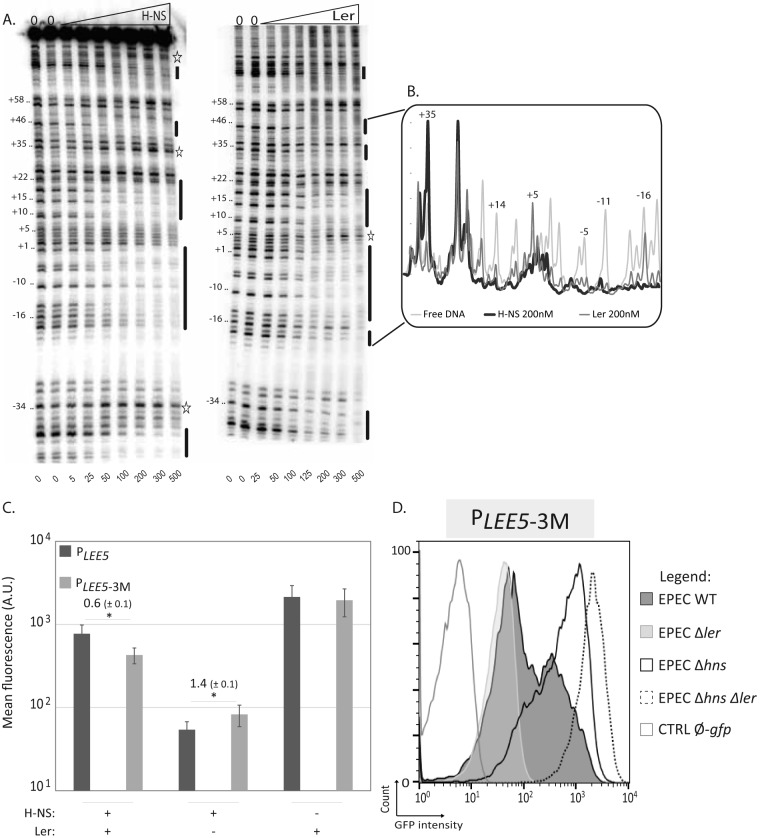
H-NS and Ler binding at the *LEE5* promoter. (A) DNase I footprints of H-NS and Ler at the *LEE5* core promoter. Increasing concentrations of H-NS or Ler were incubated with the promoter labeled on the coding strand (−80; +104) at 20°C. The nanomolar concentrations of H-NS and Ler are indicated below the lanes. The lanes with 0 above the lanes denote reactions without protein. Black bars indicate the positions of H-NS or Ler protection. The stars indicate DNase I-hypersensitive sites in the presence of H-NS or Ler. These results are representative of at least 3 independent experiments. (B) Densitometry profile of the +37 to −18 region. The profile corresponds to the DNase I footprints obtained with no protein or with 200 nM H-NS or Ler. The results were normalized to the results for the band at position +39, which does not vary with increasing protein concentration. (C) Flow cytometry analysis of *LEE5* promoter activity in EPEC strains harboring native or mutated *LEE5* promoters under nonoptimal conditions. The WT EPEC, Δ*ler*, and Δ*hns* isogenic strains containing the pKK-*P*_*LEE5*_-*gfp* (*P*_*LEE5*_) plasmid promoter or its derivative (*P*_*LEE5*-3M_) mutated at sites centered at positions −110, +15, and +254 were cultured at 37°C in LB medium. In the stationary phase (24 h), the mean fluorescence of the entire bacterial population was determined using flow cytometry analysis. The results correspond to the means ± standard errors from seven independent experiments. Significant differences (*P* < 0.05) between the values for strains with native and mutated promoters by Student’s two-paired *t* test are indicated by an asterisk. Of note, although error bars overlap because of the variation between independent experiments, a statistical difference between native and mutated promoters was reproducibly observed in the absence of Ler. The value indicated above the bars corresponds to the mean ratio ± standard error of *gfp* expression under the control of *P*_*LEE5*-3M_ over *P*_*LEE5*_ promoters. (D) Pattern of *P*_*LEE5*-3M_ activity under nonoptimal conditions. WT EPEC, Δ*ler*, and Δ*hns* single or double mutant strains containing the pKK-*P*_*LEE5*-3M_*-gfp* plasmid were cultured under agitation at 37°C in LB media. The WT EPEC strain containing the pKK-*gfp* plasmid (CTRL Ø-*gfp*) was used as a negative control. In stationary phase (24 h), GFP expression was determined by flow cytometric analysis. Results are from one experiment representative of at least three independent experiments.

To assess the molecular mechanisms underlying the effects of H-NS and Ler, we compared the binding of these two proteins to the *P*_*LEE5*_ region. We performed DNase I footprinting in the *P*_*LEE5*_ core region extending from positions −80 to +104 (Fig. 4 and Fig. S4). The overall binding pattern showed that the two proteins protected similar areas (indicated as black bars to the right of the gel), consistent with the work of Shin (48). However, significant differences between the H-NS and Ler footprints were observed at RNA polymerase-binding sites (located between positions −55 and +20) (49). Each protein induced distinct effects at positions +35, +5, −16 (Fig. 4B), and −34 (Fig. 4A). At position +35, H-NS-induced DNase I hypersensitivity and Ler protection were observed. In contrast, at positions +5 and −34, protection by H-NS and Ler-induced hyperreactivity were observed (Fig. 4A and B). As DNase I-hypersensitive sites are typically indicative of a bent or kinked local DNA structure, these results suggest different constraints on the path of the DNA double helix upon H-NS or Ler binding in this central region of the promoter. These results indicate that the fine structure of the promoter is different in the presence of Ler or H-NS, including the RNA polymerase-binding site.

10.1128/mBio.00773-17.4 FIG S4  Sequence of the *LEE5* −243 to +273 region. Nucleotide positions are defined with +1 referring to the transcription start site of *P*_*LEE5*_(C. Sanchez-SanMartin, V. H. Bustamante, E. Calva, and J. L. Puente, J Bacteriol 183:2823-2833, 2001). The −10 and −35 promoter sequences are underlined in black. The H-NS consensus binding sites, identified by PRODORIC database with their scores, are indicated in the sense (blue) or antisense (yellow) orientation. The mutations introduced at positions −110, +15 and +254 to generate *P*_*LEE5*-3M_ are indicated by the magenta boxes. The gray box indicates the position of the putative CpxR-binding site (GTAAN_6−7_GTAA). H-NS and Ler footprint-protected regions are indicated by green and brown lines, respectively (Fig. 4 and Fig. S5 and S6). Arrows indicate DNase I-hypersensitive sites. Download FIG S4, PDF file, 0.4 MB. Copyright © 2017 Leh et al. 2017 Leh et al. This content is distributed under the terms of the Creative Commons Attribution 4.0 International license.

In the present study, H-NS was found to cover a larger region of* P*_*LEE5*_ than Ler (Fig. S5). A comparable observation was previously reported, with H-NS covering larger DNA regions *in vitro* than Ler at the *lpf1* promoter (50). This finding suggests that Ler binding may not spread along DNA as much as H-NS, which typically covers up to 1,500 bp (24).

10.1128/mBio.00773-17.5 FIG S5  Comparison of H-NS and Ler binding on the extended *LEE5* promoter. (A and B) DNase I footprints of H-NS and Ler on *P*_*LEE5*_ (−228; +273) on the coding strand (A) and on the noncoding strand (B). Experiments were performed at 20°C with increasing H-NS and Ler concentrations. Black bars indicate positions of H-NS or Ler protection. Stars indicate DNase I hyperreactive sites in the presence of H-NS or Ler. Concentrations of H-NS and Ler (in nanomolar) are indicated below each lane. The “0” above the lanes denotes reactions without any protein. These results are from one experiment representative of at least three independent experiments. Nearby discrete sites are observed along the −20 to −199 region (see summary in Fig. S4). Strikingly, footprints between positions −180 and −100 show that Ler and H-NS occupy the same exact positions. However, for regions upstream of −180 and downstream of +135, only H-NS is bound. Download FIG S5, PDF file, 2 MB. Copyright © 2017 Leh et al. 2017 Leh et al. This content is distributed under the terms of the Creative Commons Attribution 4.0 International license.

H-NS binding to DNA is initiated at the level of consensus sequences (51, 52). The *P*_*LEE5*_ region displays nine consensus sequences at positions −195 to −190, −160 to −150 (2 sites), −123 to −105 (2 sites), +10 to +20, +75 to +85, +250 to +270 (2 sites) (Fig. S4). When their consensus scores were compared using Virtual footprint software (53), the best predicted site was centered at position +5, the second site was located at position −110, and the third site was located at position +254 (Fig. S4). These three sites were simultaneously disrupted by substituting the central AT-rich motif with a CG-rich motif. In the resulting promoter, referred to as “*P*_*LEE5*-3M_,” these mutations altered the binding of both H-NS and Ler *in vitro* (Fig. S6). *In vivo*, examination of the expression of *P*_*LEE5*-3M_ showed that the altered binding of both H-NS and Ler resulted in weaker silencing by H-NS and weaker antisilencing by Ler compared with the native *P*_*LEE5*_ (Fig. 4C). Consequently, when measured at the individual cell level, the two bacterial subpopulations (low and high states) are closer to each other (Fig. 4D).

10.1128/mBio.00773-17.6 FIG S6  DNase I footprints of H-NS on *P*_*LEE5*_ and *P*_*LEE5*-3M_ promoters (coding strand of the region from positions −228 to +273) at 20°C with increasing concentrations of H-NS or Ler. Concentrations of H-NS and Ler (in nanomolar) are indicated below the lanes. The 0 above the lanes denotes reactions without protein. Dashed bars indicate regions where protection is affected by the mutations. These results are representative of at least three independent experiments. Footprints were obtained on *P*_*LEE5*_ and *P*_*LEE5*-3M_ in the presence of Ler or H-NS. The *K*_*d*_ (dissociation constant) values for different sites on the native promoter range from 4 to 60 nM for both H-NS and Ler (values obtained by quantification of band intensities). For *P*_*LEE5*-3M_, each binding site (even if not mutated) displays a significant impaired affinity (all *K*_*d*_ values are superior to 100 nM) for both H-NS and Ler. These results show that the Ler binding affinity is strongly affected by the modification of important TA steps at the center of H-NS consensus sites. Download FIG S6, PDF file, 2.8 MB. Copyright © 2017 Leh et al. 2017 Leh et al. This content is distributed under the terms of the Creative Commons Attribution 4.0 International license.

Taken together, these results indicate that H-NS and Ler recognize the same or a very similar nucleation DNA motif, but H-NS induces different DNA structural changes at the RNA polymerase-binding site and covers a longer DNA region than Ler. This suggests that the roles of Ler and H-NS in the bimodal expression of *LEE5* involve competitive binding and distinctive modifications of local DNA structure organization.

### *LEE5* expression is finely tuned by Ler.

**FIG 5:**
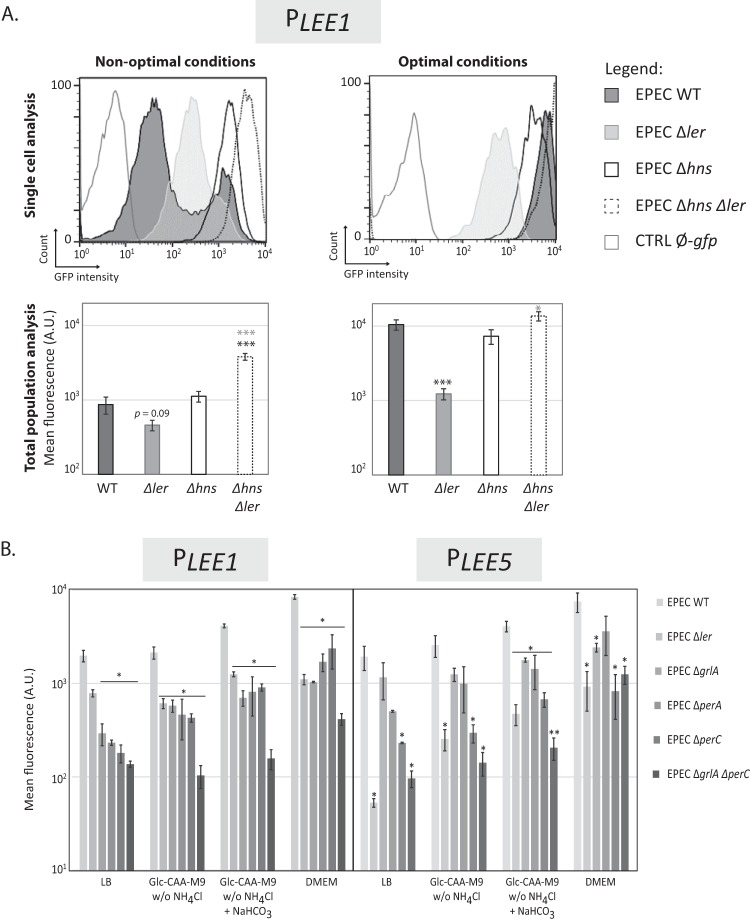
*LEE1* promoter activity presents a bimodal pattern under nonoptimal conditions as found with the *LEE5* promoter. (A) Flow cytometry analysis of *LEE1* promoter activity in EPEC strains under nonoptimal and optimal conditions. The WT EPEC, Δ*ler*, Δ*hns* single and double mutant strains containing the pKK-*P_LEE1_-gfp* plasmid were cultured at 37°C in LB (nonoptimal conditions) and DMEM (optimal conditions) media. The WT EPEC strain containing the pKK-*gfp* plasmid (CTRL Ø-*gfp*) was used as a negative control. (Top) GFP expression was determined using flow cytometry analysis at 24 h in stationary phase. The results of single-cell analysis correspond to one representative experiment (with all experiments carried out using the same settings shown in the top panels). (Bottom) Average quantification of the corresponding conditions represented in the top panels. Distinct acquisition settings were used depending on the level of cell fluorescence. The results are comparable, and all values were normalized to the mean fluorescence of the entire bacterial population (± standard error). A total of nine or four independent experiments were conducted in nonoptimal or optimal conditions, respectively. Values that are significantly different from the values for the WT strain and for the Δ*hns* single mutant by Student’s two-paired *t* test are indicated by black and gray asterisks, respectively: *, *P* < 0.05; ***, *P* < 0.001. (B) Flow cytometry analysis of *LEE1* and *LEE5* promoter activity in a *ler*, *grlA*, *perA*, or *perC* mutant background in various media. Strains containing the pKK-*P_LEE1_-gfp* or pKK-*P_LEE5_-gfp* plasmid were cultured at 37°C in DMEM, LB, and Glc-CAA-M9 without NH_4_Cl or supplemented with 45 mM NaHCO_3_ where indicated. In stationary phase, the mean fluorescence of individual bacteria was determined using flow cytometry. Distinct acquisition settings were used depending on cell fluorescence. The results are comparable, as all the values were normalized to the mean fluorescence of the entire bacterial population. Values are means ± standard errors from two independent experiments. Values that are significantly different for the WT strain and its isogenic mutant strain by Student’s one-paired *t* test are indicated with asterisks as follows: *, *P* < 0.05; **, *P* < 0.01.

We next explored variations in expression by measuring the fluorescence of cells harboring the *gfp* gene under the control of the *LEE1* promoter (“*P*_*LEE1*_”). For *P*_*LEE1*_, similar to the results with *P*_*LEE5*_, we observed a bimodal pattern of expression under nonoptimal conditions (LB) and a unimodal distribution of highly expressing cells under optimal conditions (DMEM) (Fig. 5A). These results are consistent with previous measurements of *ler* promoter expression using a chromosomal *ler-gfp* transcriptional fusion (36).

At the whole-population level, *P*_*LEE1*_ activity, like *P*_*LEE5*_ activity, varied depending on the presence or absence of either Ler or H-NS. *LEE1* expression was reduced in the absence of Ler (regardless of the medium) and increased in the absence of H-NS under nonoptimal conditions (LB). Under optimal conditions (DMEM), the H-NS silencing of *P*_*LEE1*_ (as observed for *P*_*LEE5*_ above) was completely relieved (Fig. 5A). These observations were confirmed by reverse transcription-quantitative PCR (RT-qPCR) (Fig. S7).

10.1128/mBio.00773-17.7 FIG S7  Analysis of *gfp*, *tir*, and *ler* transcript levels depending on growth conditions. WT EPEC and Δ*hns* isogenic strains containing the pKK-*P*_*LEE5*_-*gfp* plasmid were cultured at 37°C in DMEM, LB, or Glc-CAA-M9 medium. (A and B) In stationary phase, quantifications of *gfp* and *tir* (A) or *ler* (B) transcripts were determined by RT-qPCR. The results are presented as the ratio of RNAs in the Δ*hns* mutant over the WT strain condition, using *gapA*, *rpoB*, and *dnaQ* transcripts as references. (C) WT EPEC strain containing the pKK-*P_LEE5_-gfp* plasmid was cultured at 37°C in DMEM, LB, or Glc-CAA-M9 medium. *tir* transcript quantification was performed by RT-qPCR on RNA extracted in exponential (3 h) or late stationary (24 h) phases. Results are represented as the ratio of RNAs in the DMEM over LB or Glc-CAA-M9 medium conditions, using *gapA*, *rpoB*, and *dnaQ* transcripts as references. The Methods for RT-qPCR experiments follow. Bacteria were grown overnight in 4 ml of Lennox LB growth medium in 15-ml conical tubes supplemented with ampicillin (100 µg/ml) at 37°C in a shaking incubator. Samples were diluted at 1:1,000 in the indicated medium. Growth cultures were stopped in exponential (3 h) or in stationary phase (24 h) by adding 4 ml of cold absolute ethanol to 4 ml or 1 ml of bacterial suspensions, respectively. Cells were harvested by centrifugation for 15 min at 3,000 × *g* and stored at −20°C. RNA was extracted by treating cells twice with an equal volume of acidic hot phenol and once with chloroform. RNAs were ethanol precipitated, air dried, and dissolved in water. RNAs were treated with DNase I (Roche) according to the manufacturer’s instructions and were further treated once with acidic hot phenol and once with chloroform, ethanol precipitated, air dried, and dissolved in water. Three hundred nanograms of RNA was reverse transcribed in a 20-µl final reaction mixture volume using Moloney murine leukemia virus (M-MuLV) reverse transcriptase (catalog no. M0368S; New England BioLabs) following the manufacturer’s instructions in the presence of a mixture of reverse primers specific to *gfp*, *tir*, *ler*, *dnaQ*, *rpoB*, and *gapA* genes (see Table S2). cDNA (0.3 to 3 ng) was used as the template for qPCR reactions with Fast SYBR green PCR master mix (Roche Applied Science) with a Light Cycler instrument (Roche Life Science). The amplification efficiencies of each probe were generated using the slopes of the standard curves obtained by 10-fold dilution series. Gene expression levels were analyzed using the relative quantification (M. W. Pfaffl, Nucleic Acids Res 29:e45, 2001). The means of three housekeeping genes (*dnaQ*, *gapA*, and*rpoB*) were used to normalize samples. Download FIG S7, PDF file, 0.2 MB. Copyright © 2017 Leh et al. 2017 Leh et al. This content is distributed under the terms of the Creative Commons Attribution 4.0 International license.

Under nonoptimal conditions, the distribution of the Δ*ler* bacterial population was unimodal, and its fluorescence level fell between the low and high states of the WT strain (Fig. 5A). These findings are consistent with previous reports and explain why either a negative effect (54, 55) or a positive effect (35) of Ler on its own promoter was previously observed depending on growth conditions.

**FIG 6:**
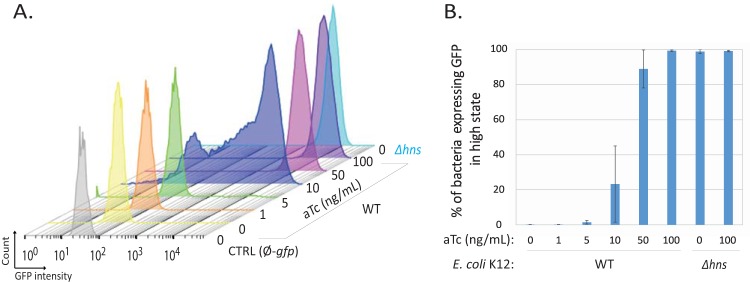
The Ler protein alone is sufficient to reconstitute the bimodal expression pattern of *LEE5* in an *E. coli* commensal strain. The WT MG1655Z1 and Δ*hns* strains expressing the TetR repressor and containing two plasmids (pKK-*P*_*LEE5*_-*gfp* and pZA31-*Ler*) were grown in LB medium at 37°C in the presence or absence of an increasing concentration of anhydrotetracycline (aTc). The WT MG1655Z1 strain containing pKK-*gfp* plasmid (CTRL Ø-*gfp*) was used as a negative control. After 24 h, cytometry analyses of bacterial populations were performed and plotted. The control strain without the *gfp* reporter (gray), WT strain with increasing aTc concentrations (yellow to dark violet), and Δ*hns* strain in the absence of aTc (turquoise blue) are shown. The results from one representative experiment (from three independent experiments) are shown in panel A. The mean (± standard error) percentages of GFP-expressing bacteria in the high state in three independent experiments are presented in panel B. In the range of 10 to 50 ng/ml aTc, where the bimodal population is observed, the proportion of bacteria in the “high state” displays high variability as indicated by the error bars.

Since GrlA, PerA, and PerC are major activators of *LEE1* expression, depending on growth conditions (36, 56), we assessed the precise roles of these activators on both *P*_*LEE1*_ and *P*_*LEE5*_ activity in the different media used here (Fig. 5B). The deletion of *ler* reduced *P*_*LEE1*_ activity but had a lower impact than the double inactivation of both *grlA* and *perC*, regardless of the medium composition (Fig. 5B). This indicates that expression from *P*_*LEE1*_ is highly dependent on these activators, while Ler plays a secondary role. As previously described (56), PerA, PerC, and GrlA independently activate *ler* expression in DMEM. Additionally, these factors were required for the optimal expression of both *LEE1* and *LEE5* in all tested media (Fig. 5B). The variation of *P*_*LEE5*_ expression therefore correlates with Ler production according to both the medium composition and the control by PerA, PerC, and GrlA. In nonoptimal conditions, *LEE1* and *LEE5* expression remained bimodal in the Δ*grlA* mutant but was unimodal and at a lower level in the Δ*perC* and Δ*grlA* Δ*perC* mutant strains (data not shown). To formally show that the Ler protein directly controls the variation of *P*_*LEE5*_ expression, we constructed a synthetic, tunable promoter (Tet-ON) controlling *ler* in the commensal *E. coli* K-12 strain. Increasing the inducer concentration resulted in a shift between the two populations expressing *LEE5* at low and high states (Fig. 6). Importantly, a bimodal pattern, with two subpopulations of cells, was also observed at an intermediary dose of the inducer (Fig. 6). Thus, in the absence of a complete *LEE* island and additional virulence factors, modulation of Ler protein levels is sufficient to induce and modulate a bimodal pattern of *LEE5* expression.

## DISCUSSION

**FIG 7:**
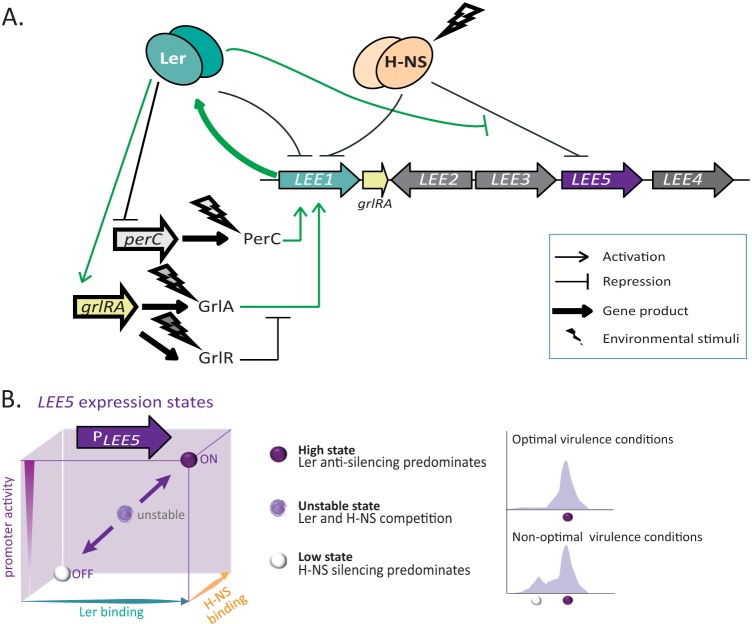
Model of *LEE5* promoter regulation that can result in a bimodal population. (A) Overview of the main regulatory networks determining *LEE1* and *LEE5* promoter expression. Environmental stimuli induce *grlA*, *perA*, and *perC* expression. H-NS acts as a repressor of *P*_*LEE1*_ and *P*_*LEE5*_. Ler directly represses its own promoter and stimulates the transcription of the *grlR-grlA* operon encoded by the LEE island. In turn, GrlA stimulates *P*_*LEE1*_. However, GrlR counteracts GrlA action (56, 57, 69). PerC, encoded by the EAF virulence plasmid, directly activates *P*_*LEE1*_ (56, 70), whereas Ler negatively regulates *perC* (35). In bacteria expressing *LEE5* at high levels, positive regulation of *P*_*LEE1*_ by PerC and GrlA predominates (green pathways). This regulatory network is analogous to a toggle switch circuit displaying high complexity, reflecting the presence of a negative-feedback loop mediated through Ler. For clarity, H-NS and Ler regulons have been limited for *LEE1*, *LEE5*, and *grlRA* operons. The LEE island is not drawn to scale. (B) *LEE5* regulation controls switch to a bimodal population. For a given bacterium in the population, the key element of *LEE5* regulation resides in the modulation of *ler* expression within the regulatory network presented in panel A. This effect results in different states (left panel). The transition from a high state (ON) to a low state (OFF) is modulated by both H-NS and Ler. The two proteins compete for mutual nucleation binding sites at the promoter. As H-NS binding increases, *P*_*LEE5*_ activity decreases (turns off), resulting in silencing of both *P*_*LEE1*_ and *P*_*LEE5*_ by H-NS (low state). As *ler* expression increases, the expression of the LEE island and associated positive-feedback loops are activated and *P*_*LEE5*_ is turned on (high state). High *ler* expression results in a high stable state as long as the positive regulatory loops persist (green circuit in panel A). Under conditions in which the *ler* expression level is not fully activated, H-NS and Ler competition results in an intermediary unstable state. At the population level, this competition generates subpopulations (right panel) (see Discussion).

In the present study, we showed that H-NS and Ler, which regulate the *LEE1* and *LEE5* promoters, are essential for generating a bimodal pattern of expression. The key parameter, depending on growth conditions, is the modulation of Ler expression (Fig. 7). Under appropriate environmental conditions (e.g., DMEM or Glc-CAA-M9* [Glc-CAA-M9 without NH_4_Cl] plus NaHCO_3_), GrlA, PerA, and PerC activate *LEE1* transcription. Moreover, Ler exerts a dual regulatory effect on its own promoter, *P*_*LEE1*_-negative autoregulation (54, 55) and/or positive indirect activation, via the stimulation of GrlA expression (57) (Fig. 5 and 7). Accordingly, at the single-cell level, the two subpopulations (in high and low states) in the WT strain merge into a single unimodal population presenting an intermediate level of *LEE1* expression in the Δ*ler* mutant (Fig. 5). Therefore, the bimodal distribution observed within a population expressing *LEE1* under nonoptimal conditions may reflect the balance between these two opposing feedback loops (Fig. 5 and 7), a type of network that has been shown to lead to bimodality (1, 58).

Moreover, we showed that stochastic expression of Ler propagates to its downstream target *LEE5*. Fluctuations in Ler levels, possibly due to the bimodal expression of the *perABC* operon (36) lead to stochastic *LEE5* expression resulting from an imbalance between Ler and H-NS levels. This imbalance manifests when bacteria are grown under nonoptimal conditions, where the quantity of Ler determines the fate of *LEE5* expression. We propose that if the concentration of Ler is sufficiently high, Ler overrides the silencing of* P*_*LEE5*_ through H-NS. Otherwise, H-NS silencing predominates (Fig. 7). High and low states of expression depend upon amplification phenomena (i.e., H-NS or Ler cooperative binding, positive-feedback loops). We also propose that in the subpopulation in a high state, a positive-feedback loop maintains Ler expression at a high level, while the second population is in a low state. In this case, H-NS repression of *P*_*LEE1*_ and *P*_*LEE5*_ predominates (Fig. 7).

The bimodal expression of *LEE5* and *LEE1* in nonoptimal conditions in this study are reminiscent of a previously described bimodal growth rate phenotype, illustrated by small and large colonies of EPEC on DMEM plates (36). Small colonies correspond to hypervirulent bacteria expressing Ler and T3SS at a high level (36). This growth rate phenotype that results in bimodality of host cell infectivity required the *per* operon but not Ler and T3SS (36). In contrast, here we show the existence of a distinctive bimodality controlling T3SS expression that requires Ler and H-NS. Therefore, these two bimodal phenotypes (growth rate [36] and T3SS expression [this study]) appear to be under the control of different regulatory mechanisms. Accordingly, in coculture experiments using WT EPEC and Δ*ler* strains, T3SS expression in the WT strain did not apparently influence fitness under the experimental conditions of the present study (see Fig. S8 in the supplemental material). Notably, the variability in various phenotypic states observed here under conditions (LB) that were nonoptimal for virulence may be advantageous for rapid adaptation to changes in environmental conditions. Consequently, the results of these *ex vivo* experiments suggest a bet-hedging strategy (1, 4, 5, 59, 60), where a small subpopulation is primed to take advantage of environmental changes. In the case of virulence, a bet-hedging strategy might bring a selective advantage by increasing the chance of successful infection or host-to-host spreading. A potential bet-hedging strategy for growth rate control is also supported as previously described by Ronin et al. (36) by the observation of large and small colonies, even after many generations of growth in nonoptimal conditions.

10.1128/mBio.00773-17.8 FIG S8  Competitive growth experiment between WT EPEC and Δ*ler* strains**.** Both WT EPEC and its isogenic Δ*ler* mutant were inoculated in a coculture at different ratios (around 25%, 50%, and 75% of Δ*ler* strain with respect to the WT) in DMEM. Twenty hours later, cultures were plated on LB agar with 25 µg/ml kanamycin or without kanamycin. The fraction of WT and Δ*ler* strains was determined from four independent experiments (represented by different colors). The linear regression equation and the coefficient of determination (*R*²) are indicated. Download FIG S8, PDF file, 0.3 MB. Copyright © 2017 Leh et al. 2017 Leh et al. This content is distributed under the terms of the Creative Commons Attribution 4.0 International license.

However, we cannot exclude the possibility that bimodal expression of *LEE1* and *LEE5* observed here might belong to a division of labor scenario, i.e., that during the course of an infection, both populations (in low and high states) may cooperate. This hypothesis may be relevant, since we observed transiently these two subpopulations in optimal conditions (DMEM and cells in exponential phase), which merged into a single upregulated population during growth of the cultures. In this case, the different phenotypes in the population may participate in specific tasks that ensure the survival of the shared genotype. Thus, the potential importance of coexisting bimodal patterns for bet-hedging or division of labor strategies for A/E pathogens remains to be further explored.

From our studies, the interplay between two proteins from the H-NS family appears to be at the heart of the stochastic gene expression regulating virulence expression. H-NS, described as a chromatin organizer protein, is highly abundant and is constitutively bound to the nucleoid (61–63). Conversely, Ler is only transiently expressed at variable levels, depending on environmental stimuli (Fig. 5), and thus, this protein could play a role as a “chromatin remodeler” of the promoter that it regulates. Notably, despite frequently being described as a fairly nonspecific protein, H-NS controls sophisticated regulatory networks in coordination with Ler, one of its paralogs. Finally, this study shows for the first time that the H-NS protein family is involved in the stochastic regulation of gene expression. Other bacterial pathogenicity islands are similarly regulated through the interplay between H-NS and antagonist proteins, such as SlyA in *Salmonella*, RovA in *Yersinia*, or ToxT in *Vibrio cholerae* (64). Future studies regarding potential bimodal expression in these organisms under specific growth conditions could provide further evidence that bacterial-chromatin structure plays an important role in the epigenetics and virulence of bacteria.

## MATERIALS AND METHODS

### Strains, plasmids, promoter fragments, and primers.

*E. coli* K-12 and the EPEC* E2348/69* strains, plasmids, and primers are listed in Tables S1 and S2 in the supplemental material. For recombinant DNA manipulation, standard techniques were used.

Promoter fragments were amplified through PCR. By convention, the promoter sequences are numbered with respect to the transcription start point (+1), with upstream and downstream locations denoted by the “–” and “+” prefixes, respectively. Fragments of the EPEC *P*_*LEE5*_ (−249 to +273) (Fig. S4) and EPEC *P*_*LEE1*_ (−257 to +285), where +1 refers to the transcription start site of the *LEE1* P1A promoter (65), were amplified from genomic DNA using the primers proLEE5S/proLEE5R and proLEE1S/proLEE1R, respectively, and subcloned into the pGEM-T Easy vector (Promega). For footprinting experiments, the extended *P*_*LEE5*_ fragment (−228 to +273) was amplified using the primers proLEE5S4 and proLEE5R. The central *P*_*LEE5*_ (−80 to +104) fragment was amplified using the primers proLEE5SVI and proLEE5RII. In all cases, the pGEM-T-Easy-*P*_*LEE5*_ construct was used as the template for PCR. To generate the mutated promoter fragment (Fig. S4), the plasmid pMRQ-*P*_LEE5-3M_ (Genart; Life Technology) was used as the template. To assay promoter activities, *P*_*LEE5*_ (−249 to +273) and *P*_*LEE1*_ (−257 to +285) fragments were cloned into pKK-*gfp* to obtain pKK-*P*_*LEE5*_-*gfp* and pKK-*P*_*LEE1*_-*gfp* (51). Notably, the pKK-*gfp* plasmid has a medium-to-low copy number (≈10 per bacterium) (44, 45). The pKK-*gfp* promoter-less* gfp* plasmid was used as a negative control to determine the basal fluorescence level of the bacteria. The phage T5 constitutive promoter (a kind gift from Pascale Boulanger), referred to as “*P2*” (Table S1), was cloned into the pKK-*gfp* plasmid using XhoI and XbaI and used as a control.

10.1128/mBio.00773-17.9 TABLE S1  Strains and plasmids used in the present study. Download TABLE S1, PDF file, 0.1 MB. Copyright © 2017 Leh et al. 2017 Leh et al. This content is distributed under the terms of the Creative Commons Attribution 4.0 International license.

We also used the *LEE5* reporter cassette as a single copy at the EPEC *attB*_Phi80_ site on the chromosome. The fragment of pKK-*P_LEE5_-gfp* containing the XmaI site and EcoRV *P_LEE5_-gfp* was subcloned into the pBBintΦ integrative base vector using the AgeI and HincII sites. Chromosomal integration with Phi80 phage integrase was performed in WT EPEC and Δ*ler* strains, as previously described (66).

Ler overexpression assays were conducted in both *E. coli* K-12 MG1655Z1 WT and Δ*hns* strains (Table S1) containing the pKK-*P_LEE5_-gfp* plasmid and the pZA31-Ler plasmid (GenScript) (Table S1).

### Purification of H-NS and Ler proteins.

The H-NS protein was purified as previously described (67), and its concentration was measured according to a previous study (61). The Ler expression plasmid was constructed from the *EPEC* genome through *ler* gene PCR amplification using the primers “Ler F3” and “Ler R3” (Table S2) and subsequently subcloned into pET 28, generating pET-*ler*. *E*. *coli* BL21(DE3)/pLysS (Invitrogen) cells were transformed with pET-*ler* and used for Ler protein overexpression. The cells were grown in LB at 37°C until reaching an optical density at 600 nm (OD_600_) of 0.8. Subsequently, protein production was induced by adding 1 mM isopropyl-β-d-thiogalactopyranoside (IPTG), and the cells were harvested 1 h later by centrifugation at 5,000 × *g* at 4°C. The cells from 1 liter of culture were resuspended in 10 ml of buffer A (20 mM phosphate, 0.5 M NaCl, 5 mM dithiothreitol [DTT], and 80 mM imidazole [pH 7]), and a cocktail of protease inhibitors (Roche) at the concentration recommended by the manufacturer. The cells were disrupted using a French press at 1,500 lb/in^2^, followed by centrifugation (18,000 × *g*, 40 min, 4°C), and the supernatant was applied to a nickel-nitrilotriacetic acid (Ni-NTA) affinity column (GE Healthcare) equilibrated with buffer A containing 80 mM imidazole. The column was subsequently washed with buffer A containing 100 mM imidazole, and the protein was eluted with buffer A containing 500 mM imidazole. The fractions were dialyzed against buffer A containing 25% glycerol, and proteins were quantified using the Bradford assay with H-NS as a standard. Aliquots were frozen in liquid N_2_ and stored at −20°C until further use. The quality of the purification was determined after SDS-PAGE analysis and staining with InstantBlue.

10.1128/mBio.00773-17.10 TABLE S2  Primers used in the present study. Download TABLE S2, PDF file, 0.1 MB. Copyright © 2017 Leh et al. 2017 Leh et al. This content is distributed under the terms of the Creative Commons Attribution 4.0 International license.

### DNase I footprinting.

Fragments were generated by PCR using one primer end labeled with [γ-^32^P]ATP (3,000 Ci mmol^−1^) and the phage T4 polynucleotide kinase (NEB). DNase I footprinting was performed after incubating a 2 to 5 nM concentration of the end-labeled promoter fragment with the proteins at the indicated concentrations at 20°C in a buffer containing 10 mM HEPES (pH 7), 50 mM K glutamate, 8 mM Mg aspartate, 4 mM DTT, 10  μg/ml of bovine serum albumin, and 0.01% NP-40. The digested products were then migrated in denaturing 7% acrylamide (19:1) gels. The analysis was performed as previously described (51).

### Media.

Bacteria were grown at 37°C in Lennox Luria-Bertani (LB) (catalog no. L3022; Sigma-Aldrich Life Science), 20 mM HEPES DMEM without phenol red (catalog no. 31053; Gibco) (containing 44 mM NaHCO_3_), SF9 (catalog no. 12548-027; Gibco) or Glc-CAA-M9 medium, corresponding to an M9 base (catalog no. 63011; Sigma-Aldrich Life Science) (containing 18.4 mM NH_4_Cl) supplemented with 2-mM magnesium sulfate, 0.1 mM calcium chloride, 1 mg/liter thiamine, 0.4% glucose, 0.5% Casamino Acids, and 50 mM 3-(*N*-morpholino)propanesulfonic acid (MOPS), pH 7.4. Where indicated, we reconstituted Glc-CAA-M9 medium without NH_4_Cl (referred to as Glc-CAA-M9*). Where appropriate, NaHCO_3_ was added at a final concentration of 45 mM.

### Flow cytometry analysis.

Bacteria were precultured overnight in 4 ml of LB supplemented with ampicillin (100 µg/ml) at 37°C under agitation. The samples were then diluted 1:1,000 in 4 ml of the appropriate medium in 15-ml conical tubes and incubated at 37°C in a shaking incubator (160 rpm, INFORS AG CH-4103). After 3 h or 24 h, single-cell fluorescence was measured using either a BD FACSCalibur (BD Biosciences) or CyFlowCube8 (Partec) flow cytometer and analyzed using FlowJo software. Bacteria harboring pKK-*gfp* were employed to calibrate appropriately the FL-1 voltage. In parallel, we measured the turbidimetry at 600 nm of each sample. We used a magnetic gate (FlowJo) selecting ≈30% of the bacterial population corresponding to the most-frequent side scatter (SSC)-forward scatter (FSC) pattern (≈10,000 events). This kind of filtering minimizes the analysis of cells differing in size and complexity that could affect the variability of fluorescence (68). The magnetic gate (FlowJo), centered on each population, allows an accurate gate on populations that may shift slightly between samples. The data were normalized to the mode and smoothed using FlowJo software.

## References

[1] Smits WK, Kuipers OP, Veening JW 2006 Phenotypic variation in bacteria: the role of feedback regulation. Nat Rev Microbiol 4:259–271. doi:10.1038/nrmicro1381. 16541134

[2] Eldar A, Elowitz MB 2010 Functional roles for noise in genetic circuits. Nature 467:167–173. doi:10.1038/nature09326. 20829787 PMC4100692

[3] Norman TM, Lord ND, Paulsson J, Losick R 2015 Stochastic switching of cell fate in microbes. Annu Rev Microbiol 69:381–403. doi:10.1146/annurev-micro-091213-112852. 26332088

[4] Ackermann M 2015 A functional perspective on phenotypic heterogeneity in microorganisms. Nat Rev Microbiol 13:497–508. doi:10.1038/nrmicro3491. 26145732

[5] Bury-Moné S, Sclavi B 18 4 2017 Stochasticity of gene expression as a motor of epigenetics in bacteria: from individual to collective behaviors. Res Microbiol doi:10.1016/j.resmic.2017.03.009. 28427910

[6] van Vliet S, Ackermann M 2015 Bacterial ventures into multicellularity: collectivism through individuality. PLoS Biol 13:e1002162. doi:10.1371/journal.pbio.1002162. 26038821 PMC4454668

[7] van Gestel J, Vlamakis H, Kolter R 2015 From cell differentiation to cell collectives: *Bacillus subtilis* uses division of labor to migrate. PLoS Biol 13:e1002141. doi:10.1371/journal.pbio.1002141. 25894589 PMC4403855

[8] Sturm A, Heinemann M, Arnoldini M, Benecke A, Ackermann M, Benz M, Dormann J, Hardt WD 2011 The cost of virulence: retarded growth of Salmonella Typhimurium cells expressing type III secretion system 1. PLoS Pathog 7:e1002143. doi:10.1371/journal.ppat.1002143. 21829349 PMC3145796

[9] Balaban NQ, Merrin J, Chait R, Kowalik L, Leibler S 2004 Bacterial persistence as a phenotypic switch. Science 305:1622–1625. doi:10.1126/science.1099390. 15308767

[10] Helaine S, Cheverton AM, Watson KG, Faure LM, Matthews SA, Holden DW 2014 Internalization of *Salmonella* by macrophages induces formation of nonreplicating persisters. Science 343:204–208. doi:10.1126/science.1244705. 24408438 PMC6485627

[11] Avraham R, Haseley N, Brown D, Penaranda C, Jijon HB, Trombetta JJ, Satija R, Shalek AK, Xavier RJ, Regev A, Hung DT 2015 Pathogen cell-to-cell variability drives heterogeneity in host immune responses. Cell 162:1309–1321. doi:10.1016/j.cell.2015.08.027. 26343579 PMC4578813

[12] Basset A, Turner KH, Boush E, Sayeed S, Dove SL, Malley R 2011 Expression of the type 1 pneumococcal pilus is bistable and negatively regulated by the structural component RrgA. Infect Immun 79:2974–2983. doi:10.1128/IAI.05117-11. 21576325 PMC3147576

[13] Zeng Q, Ibekwe AM, Biddle E, Yang CH 2010 Regulatory mechanisms of exoribonuclease PNPase and regulatory small RNA on T3SS of Dickeya dadantii. Mol Plant Microbe Interact 23:1345–1355. doi:10.1094/MPMI-03-10-0063. 20831411

[14] Czechowska K, McKeithen-Mead S, Al Moussawi K, Kazmierczak BI 2014 Cheating by type 3 secretion system-negative *Pseudomonas aeruginosa* during pulmonary infection. Proc Natl Acad Sci U S A 111:7801–7806. doi:10.1073/pnas.1400782111. 24821799 PMC4040582

[15] Diard M, Garcia V, Maier L, Remus-Emsermann MN, Regoes RR, Ackermann M, Hardt WD 2013 Stabilization of cooperative virulence by the expression of an avirulent phenotype. Nature 494:353–356. doi:10.1038/nature11913. 23426324

[16] Ackermann M, Stecher B, Freed NE, Songhet P, Hardt WD, Doebeli M 2008 Self-destructive cooperation mediated by phenotypic noise. Nature 454:987–990. doi:10.1038/nature07067. 18719588

[17] LaRock DL, Chaudhary A, Miller SI 2015 *Salmonellae* interactions with host processes. Nat Rev Microbiol 13:191–205. doi:10.1038/nrmicro3420. 25749450 PMC5074537

[18] Elliott SJ, Wainwright LA, McDaniel TK, Jarvis KG, Deng YK, Lai LC, McNamara BP, Donnenberg MS, Kaper JB 1998 The complete sequence of the locus of enterocyte effacement (LEE) from enteropathogenic *Escherichia coli* E2348/69. Mol Microbiol 28:1–4. doi:10.1046/j.1365-2958.1998.00783.x. 9593291

[19] McDaniel TK, Kaper JB 1997 A cloned pathogenicity island from enteropathogenic *Escherichia coli* confers the attaching and effacing phenotype on E. coli K-12. Mol Microbiol 23:399–407. doi:10.1046/j.1365-2958.1997.2311591.x. 9044273

[20] Umanski T, Rosenshine I, Friedberg D 2002 Thermoregulated expression of virulence genes in enteropathogenic *Escherichia coli*. Microbiology 148:2735–2744. doi:10.1099/00221287-148-9-2735. 12213920

[21] Ali SS, Xia B, Liu J, Navarre WW 2012 Silencing of foreign DNA in bacteria. Curr Opin Microbiol 15:175–181. doi:10.1016/j.mib.2011.12.014. 22265250

[22] Navarre WW, Porwollik S, Wang Y, McClelland M, Rosen H, Libby SJ, Fang FC 2006 Selective silencing of foreign DNA with low GC content by the H-NS protein in *Salmonella*. Science 313:236–238. doi:10.1126/science.1128794. 16763111

[23] Grainger DC 2016 Structure and function of bacterial H-NS protein. Biochem Soc Trans 44:1561–1569. doi:10.1042/BST20160190. 27913665

[24] Kahramanoglou C, Seshasayee AS, Prieto AI, Ibberson D, Schmidt S, Zimmermann J, Benes V, Fraser GM, Luscombe NM 2011 Direct and indirect effects of H-NS and Fis on global gene expression control in *Escherichia coli*. Nucleic Acids Res 39:2073–2091. doi:10.1093/nar/gkq934. 21097887 PMC3064808

[25] Rimsky S, Travers A 2011 Pervasive regulation of nucleoid structure and function by nucleoid-associated proteins. Curr Opin Microbiol 14:136–141. doi:10.1016/j.mib.2011.01.003. 21288763

[26] Amit R, Oppenheim AB, Stavans J 2003 Increased bending rigidity of single DNA molecules by H-NS, a temperature and osmolarity sensor. Biophys J 84:2467–2473. doi:10.1016/S0006-3495(03)75051-6. 12668454 PMC1302812

[27] Dame RT, Luijsterburg MS, Krin E, Bertin PN, Wagner R, Wuite GJ 2005 DNA bridging: a property shared among H-NS-like proteins. J Bacteriol 187:1845–1848. doi:10.1128/JB.187.5.1845-1848.2005. 15716456 PMC1064010

[28] Lim CJ, Kenney LJ, Yan J 2014 Single-molecule studies on the mechanical interplay between DNA supercoiling and H-NS DNA architectural properties. Nucleic Acids Res 42:8369–8378. doi:10.1093/nar/gku566. 24990375 PMC4117784

[29] Song D, Loparo JJ 2015 Building bridges within the bacterial chromosome. Trends Genet 31:164–173. doi:10.1016/j.tig.2015.01.003. 25682183

[30] Grainger DC, Hurd D, Goldberg MD, Busby SJ 2006 Association of nucleoid proteins with coding and non-coding segments of the *Escherichia coli* genome. Nucleic Acids Res 34:4642–4652. doi:10.1093/nar/gkl542. 16963779 PMC1636352

[31] Lucchini S, Rowley G, Goldberg MD, Hurd D, Harrison M, Hinton JC 2006 H-NS mediates the silencing of laterally acquired genes in bacteria. PLoS Pathog 2:e81. doi:10.1371/journal.ppat.0020081. 16933988 PMC1550270

[32] Oshima T, Ishikawa S, Kurokawa K, Aiba H, Ogasawara N 2006 Escherichia coli histone-like protein H-NS preferentially binds to horizontally acquired DNA in association with RNA polymerase. DNA Res 13:141–153. doi:10.1093/dnares/dsl009. 17046956

[33] Fang FC, Rimsky S 2008 New insights into transcriptional regulation by H-NS. Curr Opin Microbiol 11:113–120. doi:10.1016/j.mib.2008.02.011. 18387844 PMC2394665

[34] Dorman CJ, Kane KA 2009 DNA bridging and antibridging: a role for bacterial nucleoid-associated proteins in regulating the expression of laterally acquired genes. FEMS Microbiol Rev 33:587–592. doi:10.1111/j.1574-6976.2008.00155.x. 19207739

[35] Bingle LE, Constantinidou C, Shaw RK, Islam MS, Patel M, Snyder LA, Lee DJ, Penn CW, Busby SJ, Pallen MJ 2014 Microarray analysis of the Ler regulon in enteropathogenic and enterohaemorrhagic *Escherichia coli* strains. PLoS One 9:e80160. doi:10.1371/journal.pone.0080160. 24454682 PMC3891560

[36] Ronin I, Katsowich N, Rosenshine I, Balaban NQ 2017 A long-term epigenetic memory switch controls bacterial virulence bimodality. Elife 6:e19599. doi:10.7554/eLife.19599. 28178445 PMC5295817

[37] Kamenšek S, Podlesek Z, Gillor O, Zgur-Bertok D 2010 Genes regulated by the *Escherichia coli* SOS repressor LexA exhibit heterogeneous expression. BMC Microbiol 10:283. doi:10.1186/1471-2180-10-283. 21070632 PMC2994835

[38] Bayramoglu B, Toubiana D, van Vliet S, Inglis RF, Shnerb N, Gillor O 2017 Bet-hedging in bacteriocin producing *Escherichia coli* populations: the single cell perspective. Sci Rep 7:42068. doi:10.1038/srep42068. 28165017 PMC5292716

[39] Kenny B, DeVinney R, Stein M, Reinscheid DJ, Frey EA, Finlay BB 1997 Enteropathogenic *E. coli* (EPEC) transfers its receptor for intimate adherence into mammalian cells. Cell 91:511–520. doi:10.1016/S0092-8674(00)80437-7. 9390560

[40] Hazen TH, Daugherty SC, Shetty A, Mahurkar AA, White O, Kaper JB, Rasko DA 2015 RNA-Seq analysis of isolate- and growth phase-specific differences in the global transcriptomes of enteropathogenic *Escherichia coli* prototype isolates. Front Microbiol 6:569. doi:10.3389/fmicb.2015.00569. 26124752 PMC4464170

[41] Leverton LQ, Kaper JB 2005 Temporal expression of enteropathogenic *Escherichia coli* virulence genes in an in vitro model of infection. Infect Immun 73:1034–1043. doi:10.1128/IAI.73.2.1034-1043.2005. 15664947 PMC546935

[42] Puente JL, Bieber D, Ramer SW, Murray W, Schoolnik GK 1996 The bundle-forming pili of enteropathogenic *Escherichia coli*: transcriptional regulation by environmental signals. Mol Microbiol 20:87–100. doi:10.1111/j.1365-2958.1996.tb02491.x. 8861207

[43] Rosenshine I, Ruschkowski S, Finlay BB 1996 Expression of attaching/effacing activity by enteropathogenic *Escherichia coli* depends on growth phase, temperature, and protein synthesis upon contact with epithelial cells. Infect Immun 64:966–973. 8641808 10.1128/iai.64.3.966-973.1996 PMC173864

[44] Christensen BB, Atlung T, Hansen FG 1999 DnaA boxes are important elements in setting the initiation mass of *Escherichia coli*. J Bacteriol 181:2683–2688. 10217754 10.1128/jb.181.9.2683-2688.1999 PMC93705

[45] Saggioro C, Olliver A, Sclavi B 2013 Temperature-dependence of the DnaA-DNA interaction and its effect on the autoregulation of dnaA expression. Biochem J 449:333–341. doi:10.1042/BJ20120876. 23092251

[46] Elliott SJ, Sperandio V, Girón JA, Shin S, Mellies JL, Wainwright L, Hutcheson SW, McDaniel TK, Kaper JB 2000 The locus of enterocyte effacement (LEE)-encoded regulator controls expression of both LEE- and non-LEE-encoded virulence factors in enteropathogenic and enterohemorrhagic *Escherichia coli*. Infect Immun 68:6115–6126. doi:10.1128/IAI.68.11.6115-6126.2000. 11035714 PMC97688

[47] Mellies JL, Barron AM, Haack KR, Korson AS, Oldridge DA 2006 The global regulator Ler is necessary for enteropathogenic *Escherichia coli* colonization of Caenorhabditis elegans. Infect Immun 74:64–72. doi:10.1128/IAI.74.1.64-72.2006. 16368958 PMC1346621

[48] Shin M 2017 The mechanism underlying Ler-mediated alleviation of gene repression by H-NS. Biochem Biophys Res Commun 483:392–396. doi:10.1016/j.bbrc.2016.12.132. 28013045

[49] Kovacic RT 1987 The 0 degree C closed complexes between *Escherichia coli* RNA polymerase and two promoters, T7-A3 and lacUV5. J Biol Chem 262:13654–13661. 3308880

[50] Rojas-López M, Arenas-Hernández MM, Medrano-López A, Martínez de la Peña CF, Puente JL, Martínez-Laguna Y, Torres AG 2011 Regulatory control of the *Escherichia coli* O157:H7 lpf1 operon by H-NS and Ler. J Bacteriol 193:1622–1632. doi:10.1128/JB.01082-10. 21278287 PMC3067649

[51] Bouffartigues E, Buckle M, Badaut C, Travers A, Rimsky S 2007 H-NS cooperative binding to high-affinity sites in a regulatory element results in transcriptional silencing. Nat Struct Mol Biol 14:441–448. doi:10.1038/nsmb1233. 17435766

[52] Lang B, Blot N, Bouffartigues E, Buckle M, Geertz M, Gualerzi CO, Mavathur R, Muskhelishvili G, Pon CL, Rimsky S, Stella S, Babu MM, Travers A 2007 High-affinity DNA binding sites for H-NS provide a molecular basis for selective silencing within proteobacterial genomes. Nucleic Acids Res 35:6330–6337. doi:10.1093/nar/gkm712. 17881364 PMC2094087

[53] Münch R, Hiller K, Grote A, Scheer M, Klein J, Schobert M, Jahn D 2005 Virtual Footprint and PRODORIC: an integrative framework for regulon prediction in prokaryotes. Bioinformatics 21:4187–4189. doi:10.1093/bioinformatics/bti635. 16109747

[54] Berdichevsky T, Friedberg D, Nadler C, Rokney A, Oppenheim A, Rosenshine I 2005 Ler is a negative autoregulator of the *LEE1* operon in enteropathogenic *Escherichia coli*. J Bacteriol 187:349–357. doi:10.1128/JB.187.1.349-357.2005. 15601719 PMC538822

[55] Yerushalmi G, Nadler C, Berdichevski T, Rosenshine I 2008 Mutational analysis of the locus of enterocyte effacement-encoded regulator (Ler) of enteropathogenic *Escherichia coli*. J Bacteriol 190:7808–7818. doi:10.1128/JB.00663-08. 18835988 PMC2583616

[56] Bustamante VH, Villalba MI, García-Angulo VA, Vázquez A, Martínez LC, Jiménez R, Puente JL 2011 PerC and GrlA independently regulate Ler expression in enteropathogenic *Escherichia coli*. Mol Microbiol 82:398–415. doi:10.1111/j.1365-2958.2011.07819.x. 21895790

[57] Barba J, Bustamante VH, Flores-Valdez MA, Deng W, Finlay BB, Puente JL 2005 A positive regulatory loop controls expression of the locus of enterocyte effacement-encoded regulators Ler and GrlA. J Bacteriol 187:7918–7930. doi:10.1128/JB.187.23.7918-7930.2005. 16291665 PMC1291265

[58] Rossi FM, Kringstein AM, Spicher A, Guicherit OM, Blau HM 2000 Transcriptional control: rheostat converted to on/off switch. Mol Cell 6:723–728. doi:10.1016/S1097-2765(00)00070-8. 11030351

[59] Boles BR, Thoendel M, Singh PK 2004 Self-generated diversity produces “insurance effects” in biofilm communities. Proc Natl Acad Sci U S A 101:16630–16635. doi:10.1073/pnas.0407460101. 15546998 PMC528905

[60] Cooper TF, Beaumont HJ, Rainey PB 2005 Biofilm diversity as a test of the insurance hypothesis. Microbiology 151:2815–2816, 2816–2818. 16151192 10.1099/mic.0.28026-0

[61] Spassky A, Rimsky S, Garreau H, Buc H 1984 H1a, an *E. coli* DNA-binding protein which accumulates in stationary phase, strongly compacts DNA *in vitro*. Nucleic Acids Res 12:5321–5340. 6379600 10.1093/nar/12.13.5321 PMC318922

[62] Ali MK, Fukumura M, Sakano K, Karita S, Kimura T, Sakka K, Ohmiya K 1999 Cloning, sequencing, and expression of the gene encoding the Clostridium stercorarium xylanase C in *Escherichia coli*. Biosci Biotechnol Biochem 63:1596–1604. doi:10.1271/bbb.63.1596. 10540748

[63] Talukder A, Ishihama A 2015 Growth phase dependent changes in the structure and protein composition of nucleoid in *Escherichia coli*. Sci China Life Sci 58:902–911. doi:10.1007/s11427-015-4898-0. 26208826

[64] Stoebel DM, Free A, Dorman CJ 2008 Anti-silencing: overcoming H-NS-mediated repression of transcription in Gram-negative enteric bacteria. Microbiology 154:2533–2545. doi:10.1099/mic.0.2008/020693-0. 18757787

[65] Bhat AP, Shin M, Choy HE 2014 Identification of high-specificity H-NS binding site in *LEE5* promoter of enteropathogenic *Escherichia coli* (EPEC). J Microbiol 52:626–629. doi:10.1007/s12275-014-3562-x. 24610333

[66] Zucca S, Pasotti L, Politi N, Cusella De Angelis MG, Magni P 2013 A standard vector for the chromosomal integration and characterization of BioBrick parts in *Escherichia coli*. J Biol Eng 7:12. doi:10.1186/1754-1611-7-12. 23663425 PMC3662617

[67] Tanaka K, Muramatsu S, Yamada H, Mizuno T 1991 Systematic characterization of curved DNA segments randomly cloned from *Escherichia coli* and their functional significance. Mol Gen Genet 226:367–376. doi:10.1007/BF00260648. 1903834

[68] Silander OK, Nikolic N, Zaslaver A, Bren A, Kikoin I, Alon U, Ackermann M 2012 A genome-wide analysis of promoter-mediated phenotypic noise in *Escherichia coli*. PLoS Genet 8:e1002443. doi:10.1371/journal.pgen.1002443. 22275871 PMC3261926

[69] Padavannil A, Jobichen C, Mills E, Velazquez-Campoy A, Li M, Leung KY, Mok YK, Rosenshine I, Sivaraman J 2013 Structure of GrlR-GrlA complex that prevents GrlA activation of virulence genes. Nat Commun 4:2546. doi:10.1038/ncomms3546. 24092262

[70] Mellies JL, Elliott SJ, Sperandio V, Donnenberg MS, Kaper JB 1999 The Per regulon of enteropathogenic *Escherichia coli*: identification of a regulatory cascade and a novel transcriptional activator, the locus of enterocyte effacement (LEE)-encoded regulator (ler). Mol Microbiol 33:296–306. doi:10.1046/j.1365-2958.1999.01473.x. 10411746

