# Supplementary material for: Bacterial-Chromatin Structural Proteins Regulate the Bimodal Expression of the Locus of Enterocyte Effacement (LEE) Pathogenicity Island in Enteropathogenic Escherichia coli
Source: mBio. 2017 Aug 8;8(4):e00773-17. doi: 10.1128/mBio.00773-17 (PMC5550750; doi:10.1128/mBio.00773-17)
Supplement: FIG S1 [file mbo004173419sf1.pdf]

Figure S1: Medium effect on a phage promoter activity in WT, Ler or H-NS-deficient EPEC strains

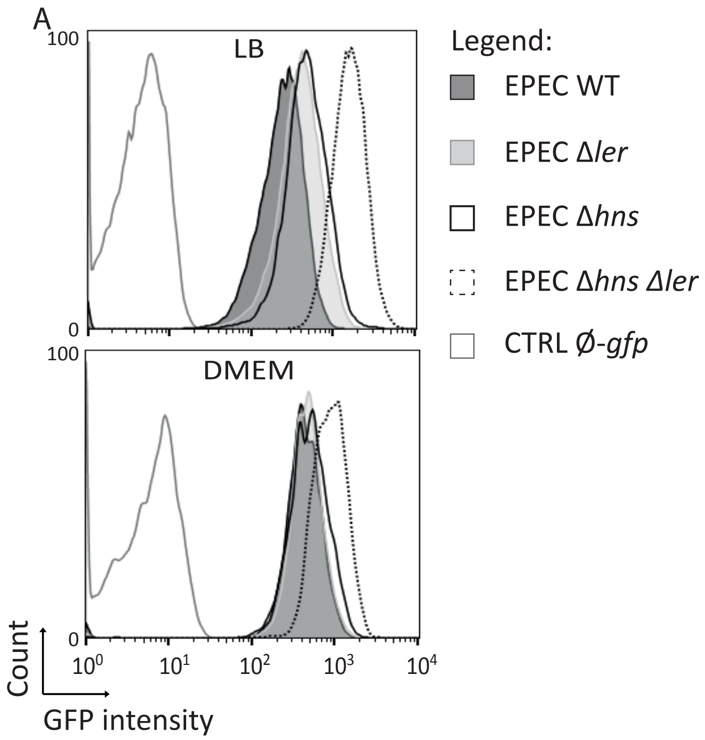

Doubling time and ratio of maximal density reached in stationary phase of EPEC strains used this study after growth in Glc-CAA-M9 medium

Strain	Maximal doubling time during exponential growth in 96-well plate reader (min) (± standard deviation, *n* = 12)	Ratio of optical density measured in stationary phase after growth in 96-well plate reader (over the WT strain, *n*=12)	Ratio of optical density measured in stationary phase after growth in 15 mL conical tube (over the WT strain, *n*=12)
EPEC WT	30.8 (± 2.2)	1	1
EPEC Δ*ler*	33.2 (± 4.3)	1.1 (± 0.1)	1.0 (± 0.1)
EPEC Δ*hns*	41.1 (± 5.9)	1.6 (± 0.3)	1.4 (± 0.2)
EPEC Δ*ler* Δ*hns*	55.0 (± 8.6)	2.1 (± 0.9)	1.7 (± 0.5)
