# Supplementary figures and images for: Bacterial-Chromatin Structural Proteins Regulate the Bimodal Expression of the Locus of Enterocyte Effacement (LEE) Pathogenicity Island in Enteropathogenic Escherichia coli
Source: mBio. 2017 Aug 8;8(4):e00773-17. doi: 10.1128/mBio.00773-17 (PMC5550750; doi:10.1128/mBio.00773-17)

**Figure S2: Kinetic of fluorescence decline by dilution by cell division**

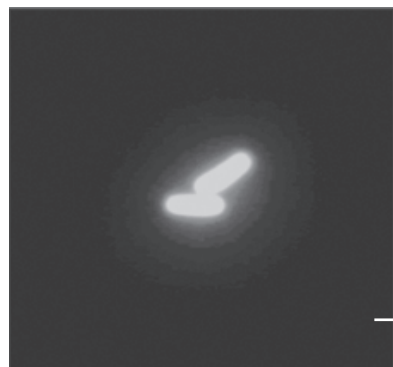

5 generations

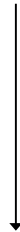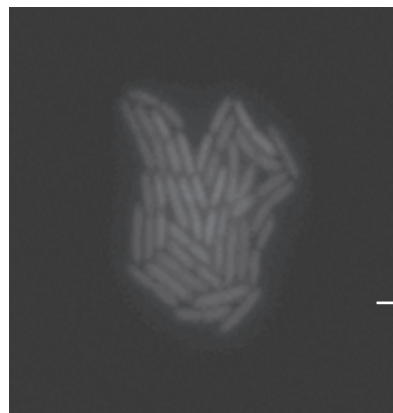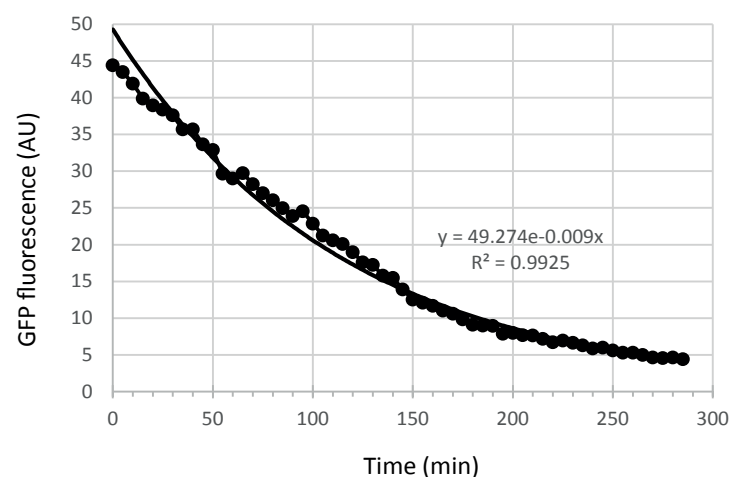

Supplement: FIG S2 [file mbo004173419sf2.pdf]

**Figure S4: Sequence of the *LEE5* -243 - +273 region**

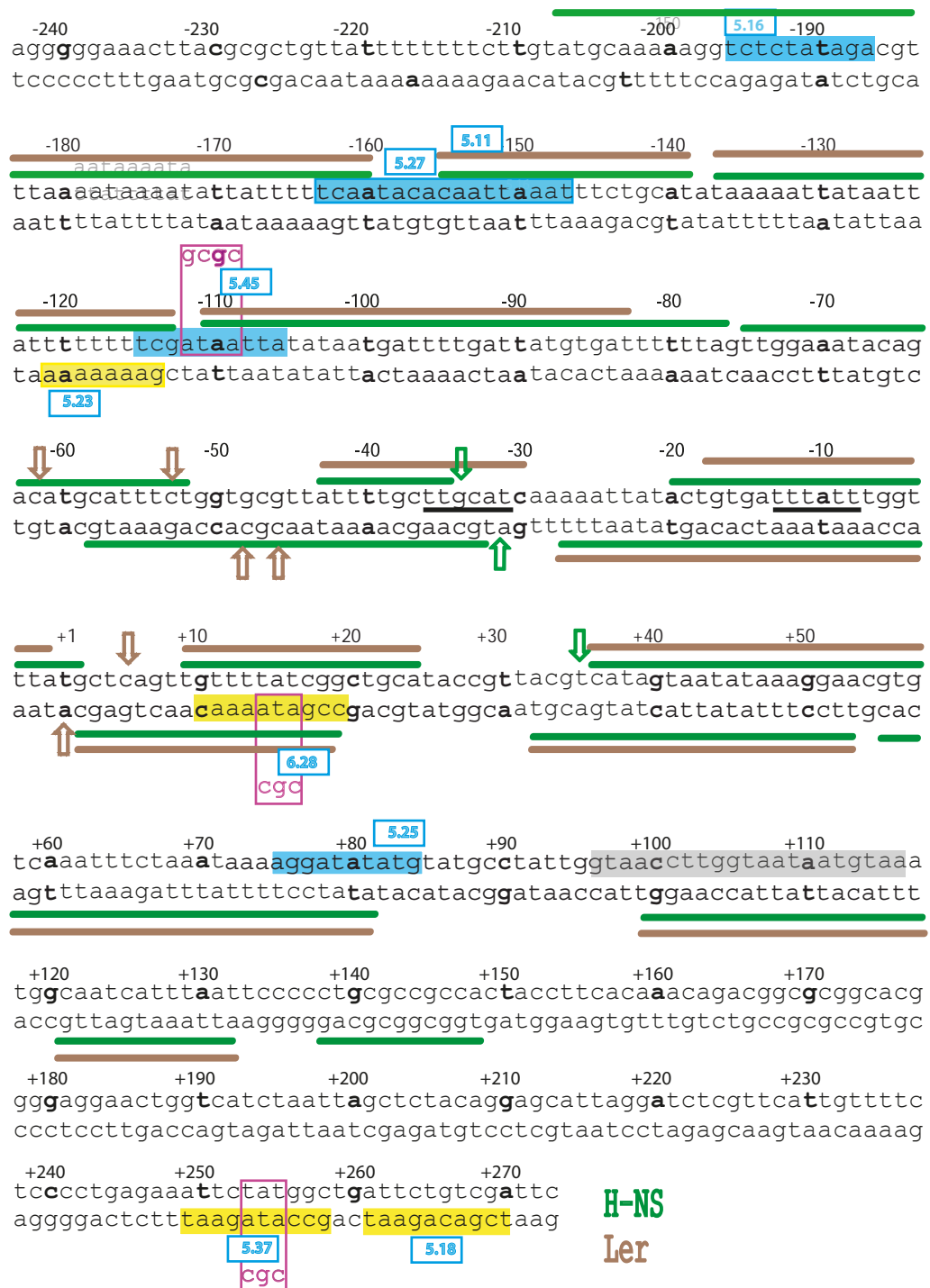

Supplement: FIG S4 [file mbo004173419sf4.pdf]

**Figure S5: Comparison of H-NS and Ler binding on the extended *LEE5* promoter**

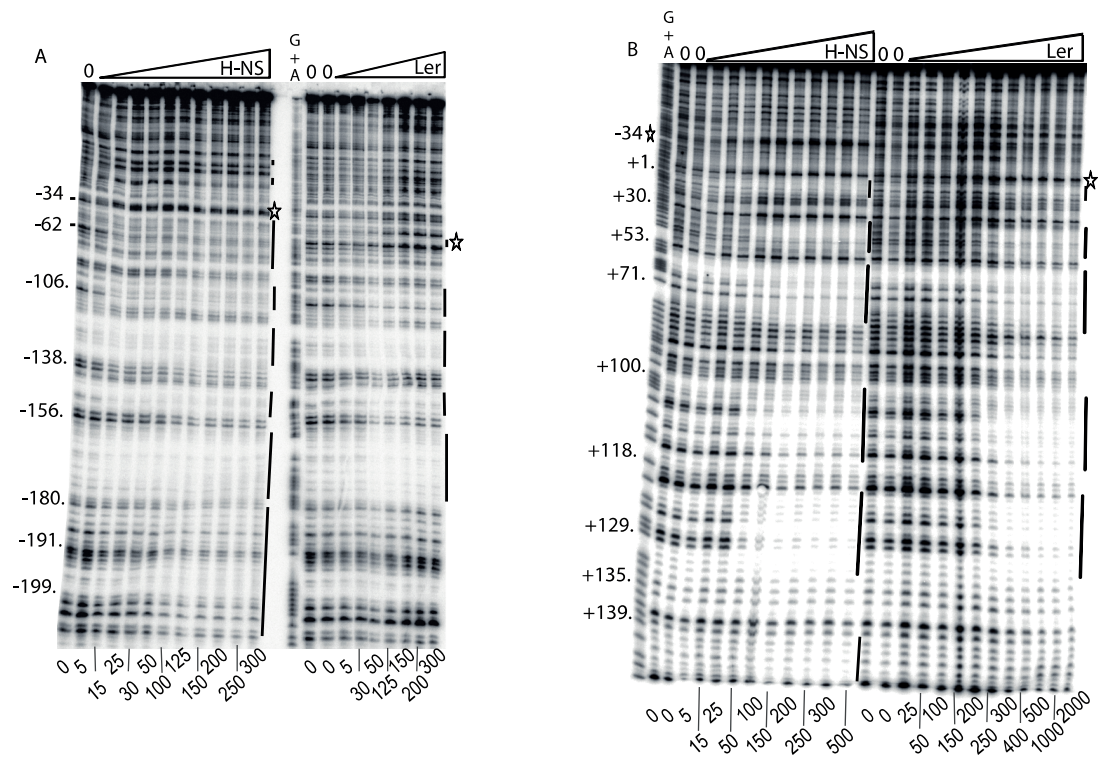

Supplement: FIG S5 [file mbo004173419sf5.pdf]

## LEE5 promoter

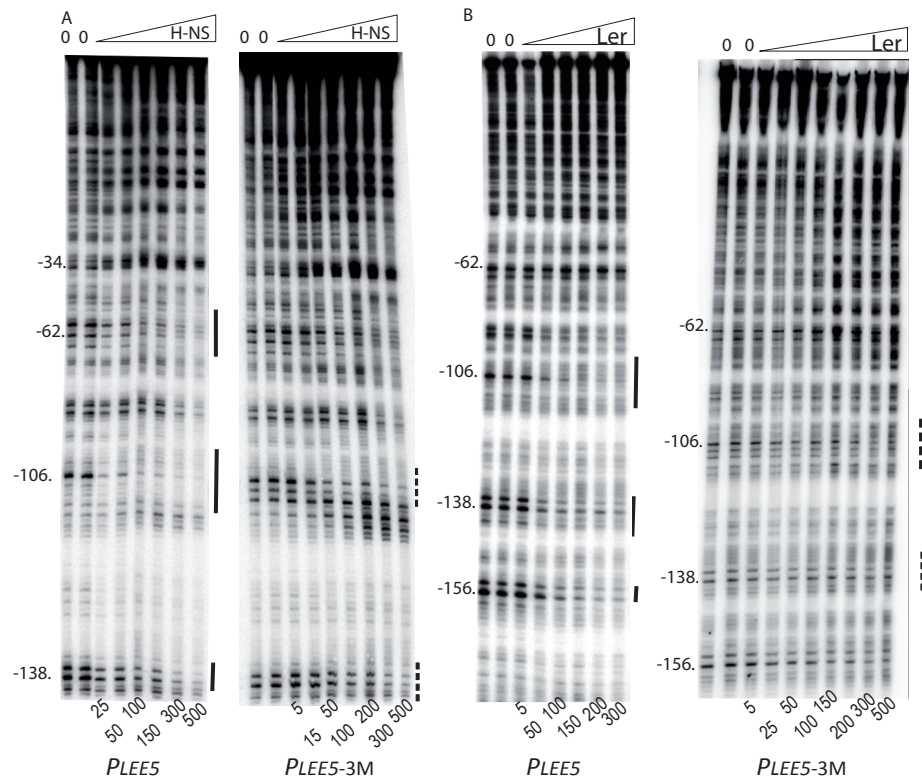

Supplement: FIG S6 [file mbo004173419sf6.pdf]

**Figure S7: Analysis of *gfp*, *tir* and *ler* transcripts level depending on growth conditions**

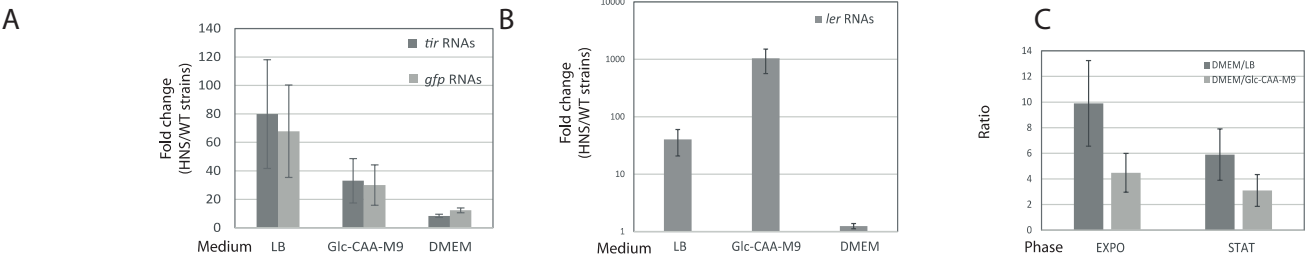

Supplement: FIG S7 [file mbo004173419sf7.pdf]

**Figure S8: Competitive growth experiment between EPEC WT and  $\Delta$ er strains**

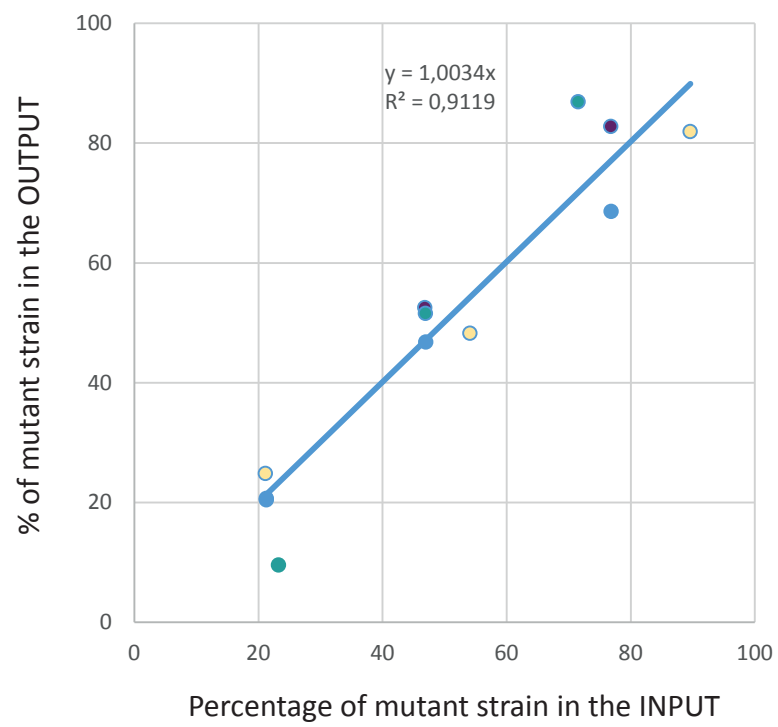

Supplement: FIG S8 [file mbo004173419sf8.pdf]
