# Supplementary material for: Bacterial-Chromatin Structural Proteins Regulate the Bimodal Expression of the Locus of Enterocyte Effacement (LEE) Pathogenicity Island in Enteropathogenic Escherichia coli
Source: mBio. 2017 Aug 8;8(4):e00773-17. doi: 10.1128/mBio.00773-17 (PMC5550750; doi:10.1128/mBio.00773-17)

Figure S3: Variation of *LEE5* expression pattern depending on the medium and growth phase of inoculating overnight culture

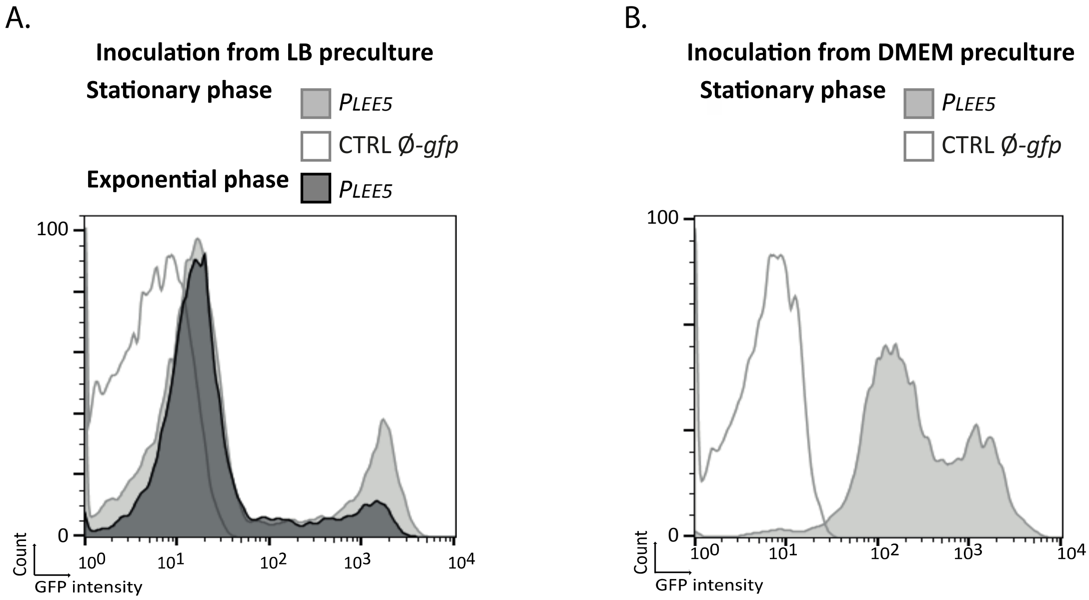

Supplement: FIG S3 [file mbo004173419sf3.pdf]
