# Supplementary material for: Bacterial-Chromatin Structural Proteins Regulate the Bimodal Expression of the Locus of Enterocyte Effacement (LEE) Pathogenicity Island in Enteropathogenic Escherichia coli
Source: mBio. 2017 Aug 8;8(4):e00773-17. doi: 10.1128/mBio.00773-17 (PMC5550750; doi:10.1128/mBio.00773-17)
Supplement: TABLE S1 [file mbo004173419st1.pdf]

Table S1: Strains and plasmids used in the present study

Name	Details	Reference or Source
**Bacterial plasmids**		
pET *ler his6*	Expression vector of the Ler_{his6} protein	This study
pGEM-T-easy Prom*LEE5*	pGEM-T with Prom*LEE5* (-250, +270) promoter region	This study
pKK-*gfp*	Derived from the pKK plasmid with promoter-less *gfpmut2* reporter fusion vector	(1)
pKK-Prom*LEE5*-*gfp*	Prom*LEE5 gfp* reporter fusion vector	This study
pKK-Prom*LEE5*-3M-*gfp*	Prom*LEE5-3M gfp* reporter fusion vector	This study
pMK-RQ-Prom*LEE5-3M*	Derived from pMK-RQ containing the Prom*LEE5* (-250, +270) sequence with the 3 disrupted H-NS binding sites	Geneart
pKK-Prom*P2*-*gfp*	T5 phage *P2* promoter *gfp* reporter fusion vector (*P2* sequence: GAGTCTGCTAATTAAACCTGAAAAACTCATTAGGTAGTTTGC TAATGGTTTTGATATAATATAA**T**AGAGATTAAATTAATAACAT AAGGA)	Sclavi's lab collection
pZA31-MCS*p*	pZ vector composed of LtetO-1 promoter (repressed by TetR repressor, induced by the addition of anhydrotetracycline), p15A replication origin and resistance to chloramphenicol	(2)
pZA31-*ler*	pZA31-MCSp vector containing *ler* gene cloned between *Kpn*I-*Bam*HI sites	This study
**Bacterial strains**		
JM109		Promega
BL21 (DE3) *pLysS*	Production of Ler_{his6}	Stratagene
E. coli MRE600	Production of native H-NS	Laboratory collection
EPEC WT	EPEC E2348/69 *wt*, $Streptomycine^R$	(3)
EPEC Δ*ler*	EPEC E2348/69 DF2 *ler::kan*	(3)
EPEC Δ*hns*	EPEC E2348/69 DF3 *hns::kan*	(3)
EPEC Δ*ler* Δ*hns*	EPEC E2348/69 TU1 *ler::kan hns ::cat*	(4)
EPEC Δ*grlA*	EPEC E2348/69 JPEP25 Δ*grlA::kan*	(5)
EPEC Δ*perA*	EPEC E2348/69 JPEP20 Δ*perA::kan*	(5)
EPEC Δ*perC*	EPEC E2348/69 JPEP22 Δ*perC::kan*	(5)
EPEC Δ*grlA* Δ*perC*	EPEC E2348/69 JPEP26 Δ*perC::kan* Δ*grlA*	(5)
MG1655Z1 WT	MG1655 containing the Z1 locus (encoding the TetR repressor	(6)

	and resistance to spectinomycin – see (2)) inserted at *att*λ site	
MG1655Z1 Δ*hns*	MG1655 *hns::kan* containing the Z1 locus inserted at *att*λ site	This study

References

1. Bouffartigues E, Buckle M, Badaut C, Travers A, Rimsky S. 2007. *Nature Structural & Molecular Biology* 14: 441–448.
2. Lutz R, Bujard H. 1997. Nucleic Acids Res 25:1203-10.
3. Friedberg D, Umanski T, Fang Y, Rosenshine I. 1999. Mol Microbiol 34:941-52.
4. Umanski T, Rosenshine I, Friedberg D. 2002. Microbiology 148:2735-44.
5. Bustamante VH, Villalba MI, Garcia-Angulo VA, Vazquez A, Martinez LC, Jimenez R, Puente JL. 2011. Mol Microbiol 82:398-415.
6. Bohn C, Collier J, Bouloc P. 2004. Mol Microbiol 52:427-35.
