# Supplementary material for: Bacterial-Chromatin Structural Proteins Regulate the Bimodal Expression of the Locus of Enterocyte Effacement (LEE) Pathogenicity Island in Enteropathogenic Escherichia coli
Source: mBio. 2017 Aug 8;8(4):e00773-17. doi: 10.1128/mBio.00773-17 (PMC5550750; doi:10.1128/mBio.00773-17)
Supplement: TABLE S2 [file mbo004173419st2.pdf]

Table S2: Primers used in the present study

Name	Sequence (5' - 3')
proLEE5 S	CGCCCGGGCTGTAGGGGGAAACTTAC
proLEE5 R	TCTCTAGAATCGACAGAATCAGCC
proLEE1 S	CGCCCGGGATTCACTCGCTTGCCGCC
proLEE1 R	CGTCTAGAACTTCCTGCTCTCGCAG
proLEE5 S4	GCGCTGTTATTTTTTTTCTTG
proLEE5 RII	CAAGGTTACCAATAGGCATAC
Pro LEE5 SVI	TTTAGTTGGAAATACAGACAT
Ler F3	GCATATGAATATGGAAACTAAT
Ler R3	CCTCGAGAATATTTTTCAGCGGTAT
Primers used for RT-qPCR:	
dnaQ-F	GTGCCGTTGAAGTGGTGAAC
dnaQ-R	GAACTCATCGGCTACCTCGG
gapA-F	CGTTGTCGCTGAAGCAACTG
gapA-R	AGCGTTGGAAACGATGTCCT
gfp-F	ACGGCCACAAGTTCTCTGTC
gfp-R	TAGTGTTGGCCATGGAACAG
ler-F	GAGAGCAGGAAGTTCAAAGTGTA
ler-R	AACACCTTTCGATGAGTTCCG
rpoB-F	TATCCGTTCCGTTGGCGAAA
rpoB-R	TGGCTGGAACCGAAGAACTC
tir-F	TCCTACAAACCCATCGAGGC
tir-R	ACCATCTGCCTGCACTTCAA
